# Developing similarity matrices for antibody-protein binding interactions

**DOI:** 10.1371/journal.pone.0293606

**Published:** 2023-10-26

**Authors:** Sumaiya Islam, Robert J. Pantazes

**Affiliations:** Department of Chemical Engineering, Auburn University, Auburn, Alabama, United States of America; Weizmann Institute of Science, ISRAEL

## Abstract

The inventions of AlphaFold and RoseTTAFold are revolutionizing computational protein science due to their abilities to reliably predict protein structures. Their unprecedented successes are due to the parallel consideration of several types of information, one of which is protein sequence similarity information. Sequence homology has been studied for many decades and depends on similarity matrices to define how similar or different protein sequences are to one another. A natural extension of predicting protein structures is predicting the interactions between proteins, but similarity matrices for protein-protein interactions do not exist. This study conducted a mutational analysis of 384 non-redundant antibody–protein antigen complexes to calculate antibody-protein interaction similarity matrices. Every important residue in each antibody and each antigen was mutated to each of the other 19 commonly occurring amino acids and the percentage changes in interaction energies were calculated using three force fields: CHARMM, Amber, and Rosetta. The data were used to construct six interaction similarity matrices, one for antibodies and another for antigens using each force field. The matrices exhibited both commonalities, such as mutations of aromatic and charged residues being the most detrimental, and differences, such as Rosetta predicting mutations of serines to be better tolerated than either Amber or CHARMM. A comparison to nine previously published similarity matrices for protein sequences revealed that the new interaction matrices are more similar to one another than they are to any of the previous matrices. The created similarity matrices can be used in force field specific applications to help guide decisions regarding mutations in protein-protein binding interfaces.

## Introduction

After decades of research, the invention of AlphaFold [1] and RoseTTAFold [2] have arguably solved the protein folding problem. Although there are important fringe cases where these methods do not yet succeed [3] and they do not work for some closely related problems [4, 5], they can correctly predict the structures of the vast majority of proteins. This will result in an increasing shift in the focus of computational protein research from structure prediction to function prediction.

Protein-protein interactions (PPIs) are crucial for biological systems to function correctly [6–9]. These interactions are complex and are influenced by many factors. Deciphering the details of PPIs requires the use of both physical chemistry analysis and observed interactions in experimentally determined protein complexes [10, 11]. Protein interfaces have been extensively studied to develop a detailed understanding of the forces and recognition processes at a molecular level [11–19]. This knowledge can be expanded by accounting for mutations, which can cause proteins’ conformations and interface properties to change [20, 21]. There are available databases of experimentally-measured changes in binding energies between proteins caused by mutations [22–24], and several computational methods have been developed to predict these changes [25–31]. A significant idea that has emerged from that prior literature is the importance of hotspot residues to PPIs, where a hotspot is a residue that is disproportionately important to a PPI.

RoseTTAFold simultaneously considers sequence alignments, distances between residues, and three-dimensional coordinates of backbone atoms in parallel tracks [2]. Similarly, the “trunk” of AlphaFold’s algorithm considers sequence alignment and residue distance information [1]. Although there are many factors that contribute to the successes of these programs, an essential component of both algorithms was the inclusion of sequence alignment information, which is based on similarity matrices. Among the oldest similarity matrices are the Point Accepted Mutation (PAM) matrices [32] and the Blocks Substitution Matrices (BLOSUM) [33], both of which have a long history of use in protein sequence alignment. Since the creation of those matrices, many other similarity matrices have been developed [34–45]. However, all of those matrices are for sequence similarity applications and we were unable to identify previously published similarity matrices for PPIs. Given the ongoing shift in emphasis to protein function prediction research, the importance of and significant prior research about PPIs, the value of similarity matrices for sequence alignment in protein structure prediction, and the lack of PPI similarity matrices, we sought to create similarity matrices for PPIs. Specifically, this work focuses on similarity matrices for PPIs in antibody-protein complexes.

While many similarity matrices have been developed in the last several decades, it is the authors’ experiences that the BLOSUM matrices in particular and the PAM matrices to a lesser extent remain the go-to starting points for similarity matrices for many researchers. This may be due to the fact that only PAM and BLOSUM matrices are selectable scoring matrices on the National Institutes of Health’s protein BLAST website [46]. As such, the PAM and BLOSUM similarity matrices were used as a conceptual reference in the development of matrices in this work. The PAM and BLOSUM matrices were created from analyses of naturally occurring protein sequences. However, two key differences guided the work in this study toward the development of computationally calculated PPI matrices. First, the analyses for PAM and BLOSUM assessed mutation rates at equivalent positions in evolutionarily related protein sequences. While there are established methods to define protein homology and thus equivalence between different positions, to the authors’ knowledge there is no established method to establish equivalency between different positions in protein interactions. As the authors were unable to identify diverse datasets of evolutionarily related PPIs to compare to one another, matrices generated from naturally occurring data could not be created. The second difference is that the effect of a mutation on the Gibbs Free Energy of a PPI is quantifiable, allowing for the determination of how a mutation changed the PPI. For these reasons, the choice was made to calculate PPI similarity matrices for the archetypical protein binding interaction, antibodies binding to protein antigens, using the CHARMM [47], Amber [48], and Rosetta [49] molecular mechanics force fields.

## Methods

### Data generation

384 antibody-protein complexes from a non-redundant database [50] were analyzed in this study. The complex from each PDB file was first minimized in CHARMM to add any missing atoms and correct conflicts between the experimental structure and the force field energy potential. The CHARMM top_all22_prot_cmap.inp topology and par_all22_prot_gbsw.inp parameter files were used for all calculations, along with the Fast Analytical Continuum Treatment of Solvation [51]. Each CHARMM minimized complex was subsequently minimized in Amber and Rosetta. The AMBER ff14SB force field [52] was used to perform the energy minimizations and calculations, with the implicit Generalized Born solvation model with surface area used with flags igb = 2 and gbsa = 1. The REF15 parameterization of Rosetta [49] was used for all calculations in Rosetta. Structures were minimized in CHARMM first as we found it was better able to resolve conflicts in the experimental structures than Amber and Rosetta.

The wild-type interaction energy of each complex was calculated according to Eq 1,

$$IE=E_{complex}-E_{Ab}-E_{Ag}$$ (1)

where *IE* is the interaction energy of the complex, *E*_*complex*_ is the force field–calculated energy of the minimized complex, *E*_*Ab*_ is the energy of the antibody alone from the minimized complex, and *E*_*Ag*_ is the energy of the antigen alone from the minimized complex. Prior analysis had revealed that the energy contributions of residues to binding in antibody-protein interfaces follow an exponential decay and that only a few residues contribute most of the binding energy [50]. Further, on average the 8^th^ most-important residue contributes less than 5% of the total binding energy in antibodies and antigens with all three force fields, while the average 7^th^ most-important residue contributes more than 5% in antibodies in all three force fields and in antigens with Rosetta. Using CHARMM and Amber, the 7^th^ most important antigen residues contribute an average of 4.9 and 4.7% of the total binding energy, respectively. Therefore, the hotspot residues were defined as the seven residues that contributed the most to binding, with both the antibodies and the antigens having their own hotspots identified. Because the energy potentials of the force fields differ from one another, the hotspot residues in each complex were determined on a force field specific basis.

Each hotspot residue in each of the complexes was mutated to each of the other 19 common amino acids. The mutations were carried out by identifying the lowest energy rotamer from a library [53] and then minimizing the energy of the complex using the same protocol as for the experimental structures. The percentage change in interaction energy (*PC*_*IE*_) for each mutant was then calculated using Eq 2, with *IE*_*Mut*_ and *IE*_*WT*_ being the interaction energies of the mutant and wild type complexes, respectively, as calculated by Eq 1.

$${PC}_{IE}=100\frac{{IE}_{Mut}-{IE}_{WT}}{{IE}_{WT}}$$ (2)

The *IE*_*WT*_ values are all negative, which is to be expected as they are closely related to the changes in Gibbs Free Energy upon binding for complexes that are experimentally proven to bind. Mutations that are predicted to improve binding correspond to those with *IE*_*Mut*_ values more negative than the *IE*_*WT*_ values. Thus, Eq 2 calculates a positive value for mutations that are predicted to improve binding and a negative value for those that are predicted to worsen it.

### Matrix calculation

There are 380 types of mutations (*i*.*e*., each of the 20 common amino acids to each of the other 19 amino acids) for each force field (*i*.*e*., CHARMM, Amber, and Rosetta) and for each protein type (*i*.*e*., antibodies and antigens). For each dataset, the median value was selected as the representative value of the mutation from amino acid *i* to amino acid *j*. Approximately 70% of the datasets exhibited non-Gaussian behavior due to the presence of extremely detrimental outliers. The presence of these outliers was expected, as it is possible to introduce mutations that cause major, irreconcilable steric clashes. The median is the preferred representation over the mean for datasets with a small number of major outliers [54].

The PAM and BLOSUM similarity matrices for protein structures were developed through different methods, but share several key similarities: they are symmetrical, their values are integers, and they have values for all entries in the matrices, including conserving the current amino acid rather than changing it. The reason the PAM and BLOSUM matrices are symmetrical arises from their comparison of known protein sequences. If protein A has amino acid X_1_ and protein B has amino acid X_2_ at equivalent positions, then it is equally valid to say that the mutation is X_1_ → X_2_ as it is to say that the mutation is X_2_ → X_1_. Thus, the number of times X_1_ mutates to X_2_ in a set of protein sequences is identical to the number of times X_2_ mutates to X_1_. For the interface mutations being studied here, that is not the case. These mutations have a direction: from an existing complex to a putative complex. A consequence of this is that the effects and scores of mutating X_1_ → X_2_ may be very different from mutating X_2_ → X_1_.

While similarity matrices for interface mutations should not be symmetrical, it is possible to generate versions that share the other features of PAM and BLOSUM. The first step in doing so is to determine appropriate numerical scores for retaining a given amino acid rather than mutating it. In PAM and BLOSUM, the scores were the percentage occurrence of each amino acid, and as a result, each row is summed to one. Here, we chose to have the percentage change in binding energy for each amino acid sum to zero. In other words, the percentage change for retaining a given amino acid was equal to the negative of the sum of all the percentage changes for mutating it.

Eq 3 was used to calculate the scores for the similarity matrices,

$$S_{i,j}=\frac{V_{i,j}}{\left|V_{i,j}\right|}round(\log_{2}\left(|V_{i,j}|+1\right))$$ (3)

where *S*_*i*,*j*_ is the score for mutating amino acid *i* to amino acid *j*, *V*_*i*,*j*_ is the representative value for mutating (or retaining) the amino acid, and *round* is the standard function of rounding a decimal number to an integer (e.g. 1.49 rounds to 1 while 1.50 rounds to 2). Logarithmic scaling was used in the BLOSUM and PAM matrices, so it was also used here. The absolute value of *V*_*i*,*j*_ was used inside the logarithm to ensure all calculated values were real and the plus one was used to guarantee that all outputs of the logarithm function were greater than or equal to 0. The leading fraction is used to assign the correct sign to the score, with beneficial mutations having positive scores and detrimental ones having negative scores.

### Matrix comparisons

Two sets of calculations were conducted to evaluate how similar two different matrices are to one another. The first is an average difference score, as calculated by Eq 4.

$$D_{m,n}=\frac{\sum_{i=1}^{20}\sum_{j=1}^{20}\left(S_{i,j,m}-S_{i,j,n}\right)}{400}$$ (4)

Here, *D*_*m1*,*m2*_ is the average difference between matrices *m* and *n*, *S*_*i*,*j*,*m*_ is the score of mutating amino acid *i* to amino acid *j* in matrix *m*, and *S*_*i*,*j*,*n*_ is the same in matrix *n*. The summation in the numerator is divided by 400 as there are 20 × 20 possible mutations when scores for retaining a particular amino acid are included. These average differences represent how much a particular matrix favors (positive) or disfavors (negative) mutations in general compared to another matrix.

The second calculation is an error calculation using Eq 5,

$$E_{m,n}=\sqrt{\frac{\sum_{i=1}^{20}\sum_{j=1}^{20}{\left(S_{i,j,m}-S_{i,j,n}-D_{m,n}\right)}^{2}}{400}}$$ (5)

where *E*_*m*,*n*_ is the square root of the average of the squared difference between the matrices of the scores for making mutations adjusted by the average difference calculated in Eq 4. The use of *D*_*m*,*n*_ in this calculation means the average difference is zero.

## Results

Table 1 shows the representative values for mutations to antibody residues as calculated by the CHARMM force field, and the results appear reasonable. The only two amino acids that exhibit a general benefit for mutation are alanine and glycine. Alanine and glycine have the smallest side chains of any amino acid: a methyl group and a hydrogen, respectively. As described in the *Methods*, the mutations were only made to the seven most important residues in an antibody for binding. If alanine and glycine are important to binding, it is likely because of contributions from their backbone atoms, which every amino acid shares. Thus, it is possible for mutations to introduce new, favorable interactions without eliminating a beneficial interaction. The inverse is true, too, that mutations could introduce detrimental interactions, but it appears that on average important alanine and glycine residues in antibodies are mutable.

**Table 1:** The representative values for mutations in antibody amino acids calculated with the CHARMM force field.

	A	C	D	E	F	G	H	I	K	L	M	N	P	Q	R	S	T	V	W	Y
A	-5.34	2.08	-6.19	-2.09	1.32	-0.17	0.81	0.56	-1.67	0.19	1.27	0.72	-0.47	0.49	-0.82	2.39	1.37	0.55	2.57	2.45
C	-2.44	67.97	-12.69	-5.94	-2.46	-3.51	0.50	-5.99	-2.70	-6.52	-6.45	-4.19	-4.10	-4.61	-2.48	1.44	-4.10	-2.13	1.58	-1.19
D	-6.93	-7.10	127.54	-2.40	-6.90	-6.72	-6.57	-6.95	-8.81	-7.32	-7.08	-6.14	-7.08	-5.93	-7.31	-7.00	-6.79	-7.06	-6.73	-6.74
E	-6.53	-6.07	-2.85	111.91	-5.14	-6.69	-5.53	-6.29	-7.12	-6.14	-5.78	-5.89	-6.34	-5.45	-6.48	-6.47	-6.39	-6.30	-5.68	-4.78
F	-6.56	-4.46	-14.26	-10.14	113.83	-6.70	-4.65	-4.23	-7.00	-4.31	-3.14	-5.95	-6.53	-4.67	-6.50	-5.58	-5.77	-4.80	-3.46	-5.12
G	-0.28	0.65	-2.55	-2.18	-0.23	-0.68	1.02	-0.11	0.01	-1.15	2.07	0.48	-2.27	0.85	2.48	-0.22	-0.20	-0.25	1.10	1.46
H	-5.48	-4.28	-8.37	-6.21	-4.05	-6.20	88.66	-3.99	-3.91	-3.99	-3.35	-4.53	-5.24	-4.65	-4.85	-4.33	-4.46	-4.33	-2.44	-4.03
I	-2.01	-0.34	-8.75	-9.40	-1.17	-3.33	-2.22	43.06	-3.66	-0.91	0.49	-2.15	-2.50	0.29	-1.32	-1.83	-2.36	-0.58	-0.61	-0.69
K	-4.95	-4.22	-6.31	-6.11	-4.44	-5.07	-4.45	-4.75	89.05	-4.74	-4.34	-4.70	-5.04	-4.44	-3.02	-4.62	-4.74	-4.75	-4.48	-3.91
L	-2.20	-0.67	-5.74	-3.39	0.14	-2.63	-0.52	-0.79	-2.43	25.88	0.27	-1.56	-1.40	-0.66	-0.18	-2.50	-1.82	-0.93	-0.26	1.39
M	-4.78	-1.48	-6.87	-8.22	-1.84	-5.38	-4.65	-1.76	-2.17	-2.85	62.38	-4.10	-3.72	-2.93	-1.76	-3.26	-2.58	-2.45	-1.36	-0.23
N	-4.36	-2.12	-5.90	-5.64	-2.69	-4.56	-2.49	-2.72	-2.82	-3.41	-2.23	62.24	-3.21	-2.45	-2.01	-3.41	-3.43	-3.54	-2.68	-2.57
P	-1.36	-0.78	-4.79	-2.39	-0.99	-2.25	-1.77	-0.98	-3.27	-0.41	-1.22	-2.48	30.61	-1.25	-2.56	-1.25	-1.18	-0.23	-0.51	-0.96
Q	-4.65	-3.83	-5.13	-5.20	-2.78	-4.97	-3.69	-3.52	-2.66	-3.06	-2.92	-3.58	-4.12	68.84	-2.07	-4.20	-4.18	-3.83	-2.15	-2.31
R	-9.63	-8.90	-11.96	-11.71	-9.13	-9.62	-9.23	-9.57	-8.06	-9.79	-8.83	-9.23	-9.70	-9.12	179.72	-9.35	-9.17	-9.46	-9.14	-8.13
S	-4.43	-2.02	-7.20	-6.44	-3.29	-5.03	-2.84	-3.35	-4.09	-3.48	-2.90	-2.27	-4.34	-2.95	-1.33	67.96	-1.64	-3.78	-3.46	-3.14
T	-3.92	-1.59	-5.11	-5.21	-2.74	-4.87	-2.21	-3.30	-3.59	-3.45	-2.44	-2.33	-4.34	-2.59	-2.06	-1.27	59.29	-3.57	-2.27	-2.45
V	-2.60	-0.75	-5.40	-4.32	-0.17	-3.42	-0.76	0.26	-1.02	-0.65	0.91	-2.10	-2.29	-1.28	-1.10	-2.55	-0.71	27.84	0.26	-0.15
W	-6.70	-6.13	-10.72	-9.43	-5.04	-7.46	-6.26	-5.34	-6.71	-5.34	-5.35	-6.69	-6.76	-6.09	-6.17	-6.83	-6.40	-6.49	125.51	-5.62
Y	-7.23	-6.71	-10.78	-9.56	-6.07	-7.71	-6.82	-6.65	-8.33	-6.83	-6.32	-7.31	-7.20	-6.79	-6.22	-7.47	-7.23	-6.89	-6.40	138.49

The table should be read as mutating from the residue at the start of a row to the amino acid at the top of a column. Alanine and glycine were the only two amino acids that showed a general benefit for mutating away from their current amino acid to another. All other amino acids preferred to remain unmutated.

Table 2 is the CHARMM-calculated similarity matrix for mutations in antibodies. The values were calculated from those in Table 1 using Eq 3. Nearly every mutation has a negative score, meaning it would be detrimental to binding. All of the antibodies being analyzed in this study are naturally occurring complexes that have been affinity-matured to bind strongly to their target antigens. It is to be expected that they should have few if any possible beneficial mutations remaining. The most positive mutation score, 2, occurs for both alanine and glycine mutations and is noticeably smaller than the smallest non-mutation score, 5, which occurs for isoleucine, leucine, proline, and valine.

**Table 2:** The CHARMM-calculated similarity matrix for antibody mutations.

	A	C	D	E	F	G	H	I	K	L	M	N	P	Q	R	S	T	V	W	Y
A	-3	2	-3	-2	1	0	1	1	-1	0	1	1	-1	1	-1	2	1	1	2	2
C	-2	6	-4	-3	-2	-2	1	-3	-2	-3	-3	-2	-2	-2	-2	1	-2	-2	1	-1
D	-3	-3	7	-2	-3	-3	-3	-3	-3	-3	-3	-3	-3	-3	-3	-3	-3	-3	-3	-3
E	-3	-3	-2	7	-3	-3	-3	-3	-3	-3	-3	-3	-3	-3	-3	-3	-3	-3	-3	-3
F	-3	-2	-4	-3	7	-3	-2	-2	-3	-2	-2	-3	-3	-3	-3	-3	-3	-3	-2	-3
G	0	1	-2	-2	0	-1	1	0	0	-1	2	1	-2	1	2	0	0	0	1	1
H	-3	-2	-3	-3	-2	-3	6	-2	-2	-2	-2	-2	-3	-2	-3	-2	-2	-2	-2	-2
I	-2	0	-3	-3	-1	-2	-2	5	-2	-1	1	-2	-2	0	-1	-2	-2	-1	-1	-1
K	-3	-2	-3	-3	-2	-3	-2	-3	6	-3	-2	-3	-3	-2	-2	-2	-3	-3	-2	-2
L	-2	-1	-3	-2	0	-2	-1	-1	-2	5	0	-1	-1	-1	0	-2	-1	-1	0	1
M	-3	-1	-3	-3	-2	-3	-2	-1	-2	-2	6	-2	-2	-2	-1	-2	-2	-2	-1	0
N	-2	-2	-3	-3	-2	-2	-2	-2	-2	-2	-2	6	-2	-2	-2	-2	-2	-2	-2	-2
P	-1	-1	-3	-2	-1	-2	-1	-1	-2	0	-1	-2	5	-1	-2	-1	-1	0	-1	-1
Q	-2	-2	-3	-3	-2	-3	-2	-2	-2	-2	-2	-2	-2	6	-2	-2	-2	-2	-2	-2
R	-3	-3	-4	-4	-3	-3	-3	-3	-3	-3	-3	-3	-3	-3	7	-3	-3	-3	-3	-3
S	-2	-2	-3	-3	-2	-3	-2	-2	-2	-2	-2	-2	-2	-2	-1	6	-1	-2	-2	-2
T	-2	-1	-3	-3	-2	-3	-2	-2	-2	-2	-2	-2	-2	-2	-2	-1	6	-2	-2	-2
V	-2	-1	-3	-2	0	-2	-1	0	-1	-1	1	-2	-2	-1	-1	-2	-1	5	0	0
W	-3	-3	-4	-3	-3	-3	-3	-3	-3	-3	-3	-3	-3	-3	-3	-3	-3	-3	7	-3
Y	-3	-3	-4	-3	-3	-3	-3	-3	-3	-3	-3	-3	-3	-3	-3	-3	-3	-3	-3	7

Most mutations are predicted to be detrimental on average, but there is a general trend of mutations becoming less detrimental when considering amino acids by groups going from charged to aromatic to polar to nonpolar. Alanine and glycine were the only amino acids that showed average benefits for being mutated rather than retained in an interface.

The charged amino acids, arginine, lysine, aspartic acid, and glutamic acid, had average mutation scores near -3, with values ranging between -3.11 for arginine and -2.53 for lysine. The aromatic amino acids, phenylalanine, tryptophan, and tyrosine, had similar average mutation scores, with values of -2.74, -3.05, and -3.05, respectively. Mutations to the polar amino acids, cysteine, asparagine, glutamine, serine, and threonine, were somewhat less detrimental, with average scores between -2.16 for glutamine and -1.79 for cysteine. Cysteine was the only charged, aromatic, or polar amino acid with any favorable mutation scores, which were one for each of histidine, serine, and tryptophan. The nonpolar amino acids, alanine, glycine, isoleucine, leucine, methionine, proline, and valine, exhibited different behavior. As already stated, alanine and glycine were the only two amino acids with negative scores for non-mutations. The average mutation for the other amino acids was detrimental, but each of them had at least one mutation that was predicted to be either neutral (i.e., a score of zero) or beneficial. For proline, these were mutations to the other nonpolar amino acids leucine and valine. Each of the other nonpolar amino acids had a neutral or beneficial mutation to an aromatic, charged, or polar amino acid.

Table 3 is the similarity matrix for mutations to important antibody residues calculated using Amber. The representative values used to create the matrix are in the S1 Table. Overall, the trends of this similarity matrix are similar to those calculated by CHARMM with individual values that are a small amount more negative. A key difference is that the highest scores for all amino acids, including alanine and glycine, are for not mutating the residue. Amber predicts that mutations to charged residues are most detrimental, with the mutation of any charged amino acid to any non-charged amino acid having a score of -4. Mutations to aromatic amino acids are also consistently disfavored with all mutations having a score of -3 except for phenylalanine to glutamic acid, which has a score of -2. Continuing the similar trends from the CHARMM matrix, mutations of polar amino acids were less disfavored than those of charged or aromatic residues while being more penalized than mutations of nonpolar amino acids. Cysteine, asparagine, serine, and threonine all have average mutation scores between -3.00 for asparagine and -2.79 for cysteine and threonine, which are approximately one point more negative than predicted by CHARMM. Histidine is a small outlier among the polar residues, as it has an average mutation score of -2.16. Histidine is also the first amino acid to have any mutations that are predicted by Amber to typically be beneficial (lysine) or neutral (glutamine). As it did with the other amino acid types, Amber predicted that the typical effect of mutations of nonpolar amino acids was more detrimental than CHARMM. However, they remain the least disfavored mutations and every nonpolar amino acid except methionine has at least one mutation to a charged or polar amino acid that has a favorable score.

**Table 3:** The Amber-calculated similarity matrix for antibody mutations.

	A	C	D	E	F	G	H	I	K	L	M	N	P	Q	R	S	T	V	W	Y
A	6	-1	-3	-2	-2	-2	-2	-2	-1	-2	-3	-2	-3	-2	2	-2	-1	-2	-2	-3
C	-3	7	-3	-3	-3	-2	-2	-3	-3	-3	-3	-3	-3	-3	-2	-2	-3	-2	-3	-4
D	-4	-4	8	-3	-4	-4	-4	-4	-4	-4	-4	-4	-4	-4	-4	-4	-4	-4	-4	-4
E	-4	-4	-3	8	-4	-4	-4	-4	-4	-4	-4	-4	-4	-4	-4	-4	-4	-4	-4	-4
F	-3	-3	-3	-2	7	-3	-3	-3	-3	-3	-3	-3	-3	-3	-3	-3	-3	-3	-3	-3
G	-2	-3	-2	0	-2	6	-2	-2	-1	-3	-2	-2	-3	-1	1	-2	-2	-3	-1	-2
H	-3	-3	-1	-2	-2	-3	6	-3	1	-2	-3	-2	-3	0	-1	-3	-3	-3	-2	-3
I	-2	-2	1	2	-1	-2	-1	5	1	-2	-2	-1	-2	0	2	-1	-1	-2	0	-1
K	-4	-4	-4	-4	-4	-4	-4	-4	8	-4	-4	-4	-4	-4	-3	-4	-4	-4	-4	-4
L	-3	-2	-2	-2	-2	-3	-2	-2	1	6	-2	-2	-2	-2	-2	-2	-2	-2	-2	-2
M	-3	-3	-2	-2	-2	-3	-2	-2	-2	-2	6	-3	-2	-3	-2	-2	-3	-2	-1	-2
N	-3	-3	-3	-3	-3	-3	-3	-3	-3	-3	-3	7	-3	-3	-3	-3	-3	-3	-3	-3
P	-2	-2	-2	-2	-3	-2	-4	-3	-1	-2	-3	-3	7	2	-3	-2	-2	-3	-3	-3
Q	-3	-3	-3	-3	-2	-3	-3	-2	-2	-3	-3	-3	-3	7	-2	-3	-3	-3	-2	-3
R	-4	-4	-4	-4	-4	-4	-4	-4	-4	-4	-4	-4	-4	-4	8	-4	-4	-4	-4	-4
S	-3	-3	-3	-3	-3	-3	-3	-3	-3	-3	-3	-2	-3	-3	-2	7	-2	-3	-3	-3
T	-3	-3	-2	-3	-3	-3	-3	-3	-2	-3	-3	-3	-3	-3	-2	-2	7	-3	-3	-3
V	-3	-2	1	-2	-2	-3	-2	-2	1	-3	-2	-1	-2	-1	-1	-2	-2	6	-2	-2
W	-3	-3	-3	-3	-3	-3	-3	-3	-3	-3	-3	-3	-3	-3	-3	-3	-3	-3	7	-3
Y	-3	-3	-3	-3	-3	-3	-3	-3	-3	-3	-3	-3	-3	-3	-3	-3	-3	-3	-3	7

The broad trends of the matrix are similar to those calculated by CHARMM in that most mutations are detrimental, mutations of charged amino acids are the most detrimental, and mutations of nonpolar amino acids are the least detrimental. A meaningful difference between Tables 2 and 3 is that Amber predicts mutations to be more detrimental than CHARMM does.

Table 4 is the similarity matrix for mutations to important antibody residues as calculated by Rosetta, with the representative values used to calculate the matrix in the S2 Table. While there are similarities between the Rosetta matrix compared to the CHARMM and Amber matrices, there are also several key differences. Mutations to the charged and aromatic residues remain consistently strongly disfavored, but the trends differ for the polar and nonpolar amino acids. Each of the nonpolar amino acids of isoleucine, leucine, methionine, and valine have only detrimental scores for mutating to any other residue and their average scores are more negative than in the other force fields. Another outlier is cysteine, which Rosetta predicts to be on average the second most unfavorable residue to mutate after tryptophan. In contrast to the other nonpolar amino acids, alanine, glycine, and proline each have at least one amino acid that is predicted to be typically beneficial to mutate the residue into. Notably, for glycine that residue is tryptophan, which represents a change from the smallest amino acid to the largest. Finally, while still detrimental on average, Rosetta’s similarity matrix assigns less punitive scores to serine mutations than CHARMM or Amber do. Of the three force fields, Rosetta is the only one to predict that any mutations of serine would typically be beneficial, which it does for phenylalanine, isoleucine, and valine.

**Table 4:** The Rosetta-calculated similarity matrix for antibody mutations.

	A	C	D	E	F	G	H	I	K	L	M	N	P	Q	R	S	T	V	W	Y
A	6	1	-3	-2	1	-2	-2	1	-2	-1	-1	-2	-2	-2	-3	-2	0	1	-1	-3
C	-3	8	-3	-3	-3	-3	-4	-4	-4	-4	-3	-4	-4	-3	-4	-4	-4	-3	-3	-4
D	-3	-3	7	-3	-3	-3	-3	-3	-4	-3	-3	-3	-3	-3	-4	-3	-3	-3	-3	-3
E	-3	-3	-3	7	-3	-3	-3	-3	-4	-3	-3	-3	-3	-3	-3	-3	-3	-3	-3	-3
F	-3	-3	-3	-3	7	-3	-3	-3	-3	-3	-3	-3	-3	-3	-3	-3	-3	-3	-3	-3
G	-1	-1	-2	-2	-1	6	-2	-2	-2	-1	-1	-2	-3	-2	-2	-2	-3	-1	1	-1
H	-2	-2	-3	-3	-1	-2	6	-1	-2	-2	-2	-2	-2	-2	-3	-2	-2	-3	-2	-2
I	-3	-3	-3	-3	-2	-3	-3	7	-3	-3	-3	-3	-3	-3	-4	-3	-3	-3	-3	-3
K	-3	-3	-3	-3	-3	-3	-3	-3	7	-3	-3	-3	-3	-3	-3	-4	-3	-3	-3	-3
L	-3	-2	-3	-3	-2	-3	-3	-2	-3	7	-3	-3	-3	-3	-3	-3	-3	-2	-3	-2
M	-3	-3	-4	-3	-3	-3	-3	-3	-3	-2	7	-3	-3	-3	-3	-3	-3	-2	-1	-3
N	-3	-3	-3	-3	-3	-3	-3	-3	-3	-2	-2	7	-3	-2	-3	-3	-3	-3	-2	-3
P	-2	1	-4	-3	-2	-2	-4	-3	-3	1	-1	-2	6	-3	-2	-2	0	-1	2	-2
Q	-3	-3	-3	-3	-2	-3	-3	-3	-3	-3	-3	-3	-3	7	-2	-3	-3	-3	-2	-2
R	-3	-3	-3	-3	-3	-3	-3	-3	-3	-3	-3	-3	-3	-3	7	-3	-3	-3	-3	-3
S	0	-1	-2	-2	1	-1	-2	1	-2	-1	-1	-1	-2	-1	-2	5	-2	1	-2	-1
T	-3	-2	-3	-3	-2	-3	-3	-2	-2	-1	-2	-3	-3	-2	-3	-3	6	-2	-3	-3
V	-2	-3	-3	-3	-2	-2	-3	-2	-3	-2	-2	-3	-3	-2	-3	-3	-3	7	-3	-3
W	-4	-3	-4	-4	-3	-4	-4	-3	-4	-3	-3	-4	-4	-4	-4	-4	-3	-3	8	-3
Y	-3	-3	-3	-3	-3	-3	-3	-3	-3	-3	-3	-3	-3	-3	-3	-3	-3	-3	-3	7

While all amino acids have positive scores for not mutating, some of the other trends differ compared to the CHARMM and Amber matrices. In particular, mutations of cysteine, isoleucine, leucine, methionine, and valine are more strongly disfavored by Rosetta compared to the other force fields. In contrast, mutations of serine are less disfavored by Rosetta, which is the only force field to predict that there are specific mutations of serine (*i*.*e*., to phenylalanine, isoleucine, and valine) that are typically beneficial.

The antibodies in the evaluated complexes have undergone an affinity maturation process to improve their binding affinities with their target antigens. In contrast, the bound proteins have not been changed to better bind to the antibodies. To explore how evolutionary direction may have impacted the similarity matrices, additional matrices were made for each force field for the antigens. Table 5 is the similarity matrix for the important residues in antigens calculated using CHARMM. The representative values used to calculate the matrix are in the S3 Table. The trends share many similarities with the matrix for the antibody mutations. Mutating the charged amino acids is consistently predicted to be the most detrimental, followed closely by the aromatic residues. Mutations of the polar amino acids are on average less detrimental than those of charged or aromatic residues, but none of the polar amino acids has any individual mutation that is on average predicted to be beneficial and only cysteine has any (to alanine, methionine, and tryptophan) that are predicted to be neutral. In contrast, every nonpolar amino acid except for leucine (three neutral mutations) and methionine (all detrimental mutations) has at least one individual mutation that is predicted to be beneficial. The most meaningful distinction among the nonpolar amino acids in the antigens versus the antibodies is that mutations of glycine are expected to be, on average, detrimental while those to valine are beneficial. On the whole, the impact of mutations on the most important antigen residues is less detrimental than those to antibodies, with the total of all scores in Table 5 being -581 versus the -635 of Table 2.

**Table 5:** The CHARMM-calculated similarity matrix for antigen mutations.

	A	C	D	E	F	G	H	I	K	L	M	N	P	Q	R	S	T	V	W	Y
A	-3	1	-3	-2	1	-1	1	1	-1	1	1	1	0	1	2	0	0	1	2	2
C	0	5	-2	-3	-2	-1	-1	-1	-2	-2	0	-1	-1	-1	-2	-1	-1	-1	0	-1
D	-3	-3	7	-2	-3	-3	-3	-3	-3	-3	-3	-3	-3	-3	-3	-3	-3	-3	-3	-3
E	-3	-3	-3	7	-3	-3	-3	-3	-3	-3	-3	-3	-3	-3	-3	-3	-3	-3	-3	-3
F	-3	-2	-3	-3	6	-3	-2	-2	-3	-2	-2	-2	-3	-2	-2	-3	-2	-2	-2	0
G	0	1	-2	-2	1	3	-1	-1	-1	1	1	0	-1	1	2	0	0	-1	0	0
H	-2	-1	-3	-3	-1	-2	6	-2	-2	-2	-1	-2	-2	-1	-2	-2	-2	-2	-2	-1
I	-2	-1	-3	-4	-1	-3	-2	6	-2	-1	0	-2	-2	1	0	-2	-2	-1	-1	-1
K	-3	-3	-3	-3	-3	-3	-3	-3	7	-3	-3	-3	-3	-3	-1	-3	-3	-3	-3	-3
L	-2	-1	-3	-3	0	-2	-1	-1	-2	5	0	-2	-2	-1	-1	-2	-1	-1	0	-1
M	-2	-2	-3	-2	-2	-3	-2	-2	-1	-2	6	-3	-2	-2	-1	-2	-2	-2	-1	-1
N	-2	-2	-3	-3	-2	-3	-2	-2	-2	-2	-2	6	-2	-2	-1	-2	-2	-2	-1	-2
P	-1	-1	-2	-2	-1	-2	-1	-1	-1	-1	0	-1	4	0	1	-1	-1	-1	2	0
Q	-3	-2	-3	-3	-2	-3	-2	-2	-3	-2	-2	-2	-3	6	-2	-3	-2	-2	-2	-2
R	-3	-3	-4	-4	-3	-3	-3	-3	-3	-3	-3	-3	-3	-3	7	-3	-3	-3	-3	-3
S	-2	-1	-3	-2	-2	-3	-1	-2	-2	-2	-2	-1	-2	-1	-1	6	-1	-2	-1	-2
T	-2	-1	-3	-3	-1	-3	-2	-2	-2	-2	-1	-2	-2	-1	-1	-1	6	-2	-1	-1
V	-1	1	-1	-1	0	-1	-1	1	0	1	1	0	-1	0	1	0	1	-3	1	2
W	-3	-3	-4	-4	-2	-3	-2	-3	-3	-2	-2	-3	-3	-2	-2	-3	-3	-3	7	-2
Y	-3	-3	-3	-3	-3	-3	-3	-3	-3	-3	-3	-3	-3	-3	-2	-3	-3	-3	-2	7

The trends compared to the CHARMM matrix for antibodies are very similar. Charged and aromatic residues are the most detrimental to mutate. Polar amino acids are the next most detrimental, while nonpolar residues are the least detrimental to mutate. The total sum of scores in this matrix is 54 points larger than that of the antibody matrix, indicating that antigen mutations are on average better tolerated than antibody mutations.

Table 6 is the similarity matrix for the important residues in antigens calculated using Amber from the representative values in the S4 Table. The trends it shows are qualitatively similar to those in Table 3. In particular, mutations of charged amino acids are the worst scoring, mutations of aromatic residues are also predicted to be detrimental, and the scores are on average more negative than the corresponding CHARMM-calculated scores. The calculated mutation scores for polar residues are also quite similar to their antibody counterparts. Interestingly, that is not the case for the nonpolar residues which are predicted to have more detrimental impacts on binding than the corresponding antibody mutations. Alanine to lysine, alanine to arginine, and proline to arginine are the only mutations that have positive mutation scores. Unlike the CHARMM matrices, the sum of all scores in this matrix is 53 points more negative than the corresponding antibody matrix (-936 versus -883).The final similarity matrix calculated in this study was for mutations to important residues in antigens using the Rosetta force field. It is shown in Table 7 and the corresponding representative values are in the S5 Table. As was the case for the mutations to important antibody residues shown in Table 4, the trends have some similarities to the CHARMM and Amber data with some striking discrepancies. As has been the case in every similarity matrix, mutations of the charged and aromatic amino acids are strongly disfavored. The average mutation score of cysteine is less negative in the antigens than the antibodies (-2.74 versus -3.53) but is still clearly disfavored. It is comparably disfavored as mutations of glutamine (-2.84) and threonine (-2.74), two of the other polar amino acids. However, asparagine and histidine have average mutation scores of -2.00 and -1.89, respectively, which are less negative than seen by any calculations with Amber or CHARMM and are also less detrimental than any nonpolar residue except glycine in this matrix. Rosetta predicts that mutations to isoleucine, leucine, methionine, and valine are all typically detrimental, while alanine and proline have neutral or favorable mutations, respectively, only to tryptophan.

**Table 6:** The Amber-calculated similarity matrix for antigen mutations.

	A	C	D	E	F	G	H	I	K	L	M	N	P	Q	R	S	T	V	W	Y
A	5	-2	-2	-2	-2	-2	-2	-2	1	-2	-2	0	-2	-2	2	-2	-2	-2	-1	0
C	-2	6	-2	-2	-2	-2	-2	-2	-1	-2	-2	-2	-3	-2	-2	-2	-2	-2	-2	-2
D	-4	-4	8	-3	-4	-4	-4	-4	-4	-4	-4	-4	-4	-4	-4	-4	-4	-4	-4	-4
E	-4	-4	-3	8	-4	-4	-4	-4	-4	-4	-4	-4	-4	-4	-4	-4	-4	-4	-4	-4
F	-3	-3	-3	-3	7	-4	-3	-3	-3	-3	-3	-3	-3	-3	-3	-3	-3	-3	-3	-3
G	-3	-2	-3	-2	-3	7	-3	-3	-2	-3	-3	-3	-3	-2	-2	-3	-3	-3	-2	-3
H	-3	-3	-2	-3	-3	-3	7	-3	-2	-3	-3	-3	-3	-3	-2	-3	-3	-3	-2	-3
I	-3	-2	-2	-3	-3	-3	-2	6	-1	-2	-2	-2	-3	-1	-1	-2	-2	-2	-3	-2
K	-4	-4	-4	-4	-4	-4	-4	-4	8	-4	-4	-4	-4	-4	-3	-4	-4	-4	-4	-4
L	-3	-3	-3	-3	-3	-3	-3	-3	-2	7	-3	-3	-3	-2	-1	-3	-3	-3	-3	-3
M	-3	-3	-3	-3	-3	-3	-3	-3	-2	-3	7	-3	-3	-3	-2	-3	-3	-3	-3	-3
N	-3	-3	-3	-3	-3	-3	-3	-3	-2	-3	-3	7	-3	-3	-2	-3	-3	-3	-3	-3
P	-3	-2	-2	-2	-2	-3	-2	-2	-1	-2	-2	-1	6	-2	2	-2	-2	-2	-2	-2
Q	-3	-3	-3	-3	-3	-3	-3	-3	-3	-3	-3	-3	-3	7	-3	-3	-3	-3	-3	-3
R	-4	-4	-4	-4	-4	-4	-4	-4	-4	-4	-4	-4	-4	-4	8	-4	-4	-4	-4	-4
S	-3	-3	-2	-2	-3	-3	-3	-3	-2	-3	-3	-2	-3	-2	-1	7	-2	-3	-3	-3
T	-3	-3	-3	-3	-3	-3	-3	-3	-2	-3	-3	-3	-3	-3	-2	-3	7	-3	-3	-3
V	-2	-2	-1	-2	-2	-2	-2	-2	-1	-2	-2	-2	-2	-1	-2	-1	-2	6	-2	-2
W	-3	-3	-3	-3	-3	-3	-3	-3	-3	-3	-3	-3	-3	-3	-3	-3	-3	-3	7	-3
Y	-3	-3	-3	-3	-3	-3	-3	-3	-3	-3	-3	-3	-3	-3	-3	-3	-3	-3	-3	7

While the trends in this matrix are very similar to those in Table 3, mutations of important nonpolar residues in antigens have worse scores and thus are typically more detrimental to binding than those for antibodies.

**Table 7:** The Rosetta-calculated similarity matrix for antigen mutations.

	A	C	D	E	F	G	H	I	K	L	M	N	P	Q	R	S	T	V	W	Y
A	6	-1	-3	-3	-1	-3	-3	-1	-3	-2	-2	-3	-2	-2	-3	-3	-2	-1	0	-2
C	-2	7	-3	-3	-1	-3	-3	-2	-4	-2	-2	-2	-3	-3	-4	-3	-3	-2	-4	-3
D	-3	-3	7	-3	-3	-3	-3	-3	-4	-3	-3	-3	-3	-3	-3	-3	-3	-3	-3	-3
E	-3	-3	-3	7	-3	-3	-3	-3	-3	-3	-3	-3	-3	-3	-3	-3	-3	-3	-3	-3
F	-3	-4	-4	-4	8	-4	-3	-3	-4	-3	-3	-3	-4	-3	-4	-4	-3	-3	-3	-3
G	2	1	-2	-2	2	4	-2	-1	-2	2	2	-2	-3	0	-2	-3	-2	1	2	0
H	-2	-2	-3	-3	-1	-2	6	-1	-2	-2	-1	-3	-2	-2	-2	-3	-2	-1	-1	-1
I	-3	-2	-3	-3	-2	-3	-3	7	-3	-2	-2	-3	-3	-3	-3	-3	-3	-2	-2	-2
K	-3	-3	-4	-3	-3	-3	-3	-3	7	-3	-3	-3	-3	-3	-3	-3	-3	-3	-3	-3
L	-3	-3	-3	-4	-3	-3	-3	-2	-4	7	-2	-3	-3	-3	-3	-4	-3	-2	-3	-3
M	-4	-4	-4	-4	-3	-4	-4	-4	-4	-3	8	-4	-4	-4	-4	-4	-4	-3	-3	-3
N	-2	-2	-3	-3	-1	-3	-3	0	-3	-1	-1	6	-2	-2	-3	-3	-2	-1	-1	-2
P	-2	-1	-3	-3	-2	-3	-3	-1	-3	-1	-1	-3	6	-3	-3	-3	-3	-1	1	-2
Q	-3	-3	-3	-3	-2	-3	-3	-3	-3	-2	-2	-3	-3	7	-3	-3	-3	-3	-3	-3
R	-3	-3	-3	-3	-2	-3	-3	-2	-3	-3	-3	-3	-3	-3	7	-3	-3	-3	-3	-3
S	-2	1	-2	-3	2	-2	-2	-1	-2	0	2	-3	-2	-2	-3	5	-2	0	0	2
T	-2	-3	-3	-3	-2	-3	-3	-2	-3	-2	-3	-3	-3	-3	-3	-3	7	-3	-2	-3
V	-2	-3	-3	-3	-2	-3	-3	-2	-3	-3	-2	-3	-3	-3	-3	-4	-3	7	-3	-3
W	-4	-4	-4	-4	-3	-4	-4	-3	-4	-3	-3	-4	-4	-4	-4	-4	-4	-4	8	-4
Y	-3	-3	-3	-3	-2	-3	-3	-3	-3	-3	-3	-3	-4	-3	-3	-3	-3	-3	-3	7

The data share similarities with Table 4. In particular, mutations of isoleucine, leucine, methionine, and valine are more detrimental than in other matrices while mutations of serine, especially to nonpolar residues, are less detrimental.

The two amino acids with multiple mutations predicted to be typically beneficial are glycine and serine. Mutations of glycine are typically beneficial when they are changes to alanine, cysteine, phenylalanine, leucine, methionine, valine, and tryptophan and are neutral when they are changes to glutamine or tyrosine. Mutations of serine are typically beneficial when they are changes to cysteine, phenylalanine, methionine, and tyrosine and are neutral when they are changes to leucine, valine, and tryptophan. Notable in those two lists are the preponderance of nonpolar and aromatic amino acids capable of making hydrophobic interactions. Overall, the sum of all scores in this similarity matrix is less detrimental than those in the antibody matrix by 40 points (-819 versus -859), which is similar to the CHARMM results.

It is of interest to quantitatively compare the six calculated antibody-protein PPI similarity matrices to one another, as well as to previously published similarity matrices for protein sequence comparisons. Nine such matrices were selected for the comparison: two each of the PAM (*i*.*e*., PAM30 and 60) and BLOSUM (*i*.*e*., BLOSUM62 and BLOSUM80) families of matrices, the matrix of Miyazawa and Jernigan based on contact frequencies in protein structures [34], the matrix of Qian and Goldstein for generating accurate alignments [41], the matrix of Saigo, Vert and Akutsu for finding distant homologues [42], the matrix of Yamada and Tomii for finding distant homologues [43], and the matrix of Jia and Jernigan for considering pairs of substitutions in densely packed globular proteins [45].

**Fig 1:**
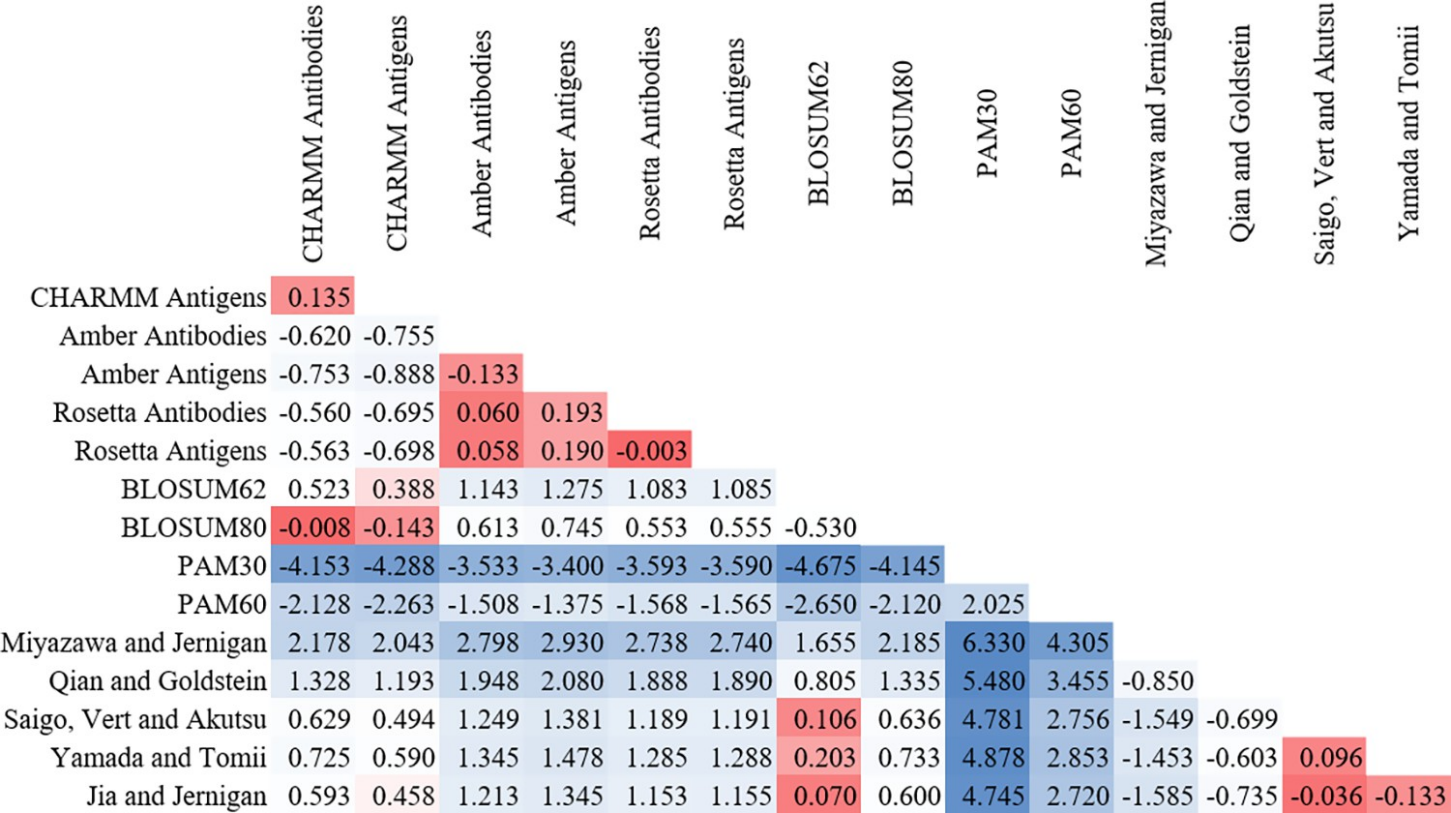
Average difference scores for similarity matrices. These values represent how much a matrix favors (positive) or disfavors (negative) mutations relative to another matrix. Numbers are colored based on their absolute values, with smaller magnitude numbers (*i*.*e*., for matrices that are more similar to one another) in red and larger magnitude numbers in blue. The antibody-protein PPI matrices calculated from the same force field (e.g., CHARMM antibodies and antigens) each have small difference scores from one another. The most notable remaining similarities are those between BLOSUM80 and the CHARMM matrices and those between the Rosetta and Amber matrices.

The average difference scores as calculated by Eq 4 are shown in Fig 1. Values on the diagonal are not shown because they would all be 0 and values above the diagonal are not shown because they are the negatives of the transposed matrix. The values show that the matrices calculated with each force field have low similarity scores. The Rosetta and Amber matrices are also similar to one another, though not with the CHARMM matrices. Almost all of the sequence similarity matrices show large average differences with the PPI matrices calculated in this work. The most notable exception is the similarity between the BLOSUM80 matrix and the CHARMM matrices, which have quite small average differences.

**Fig 2:**
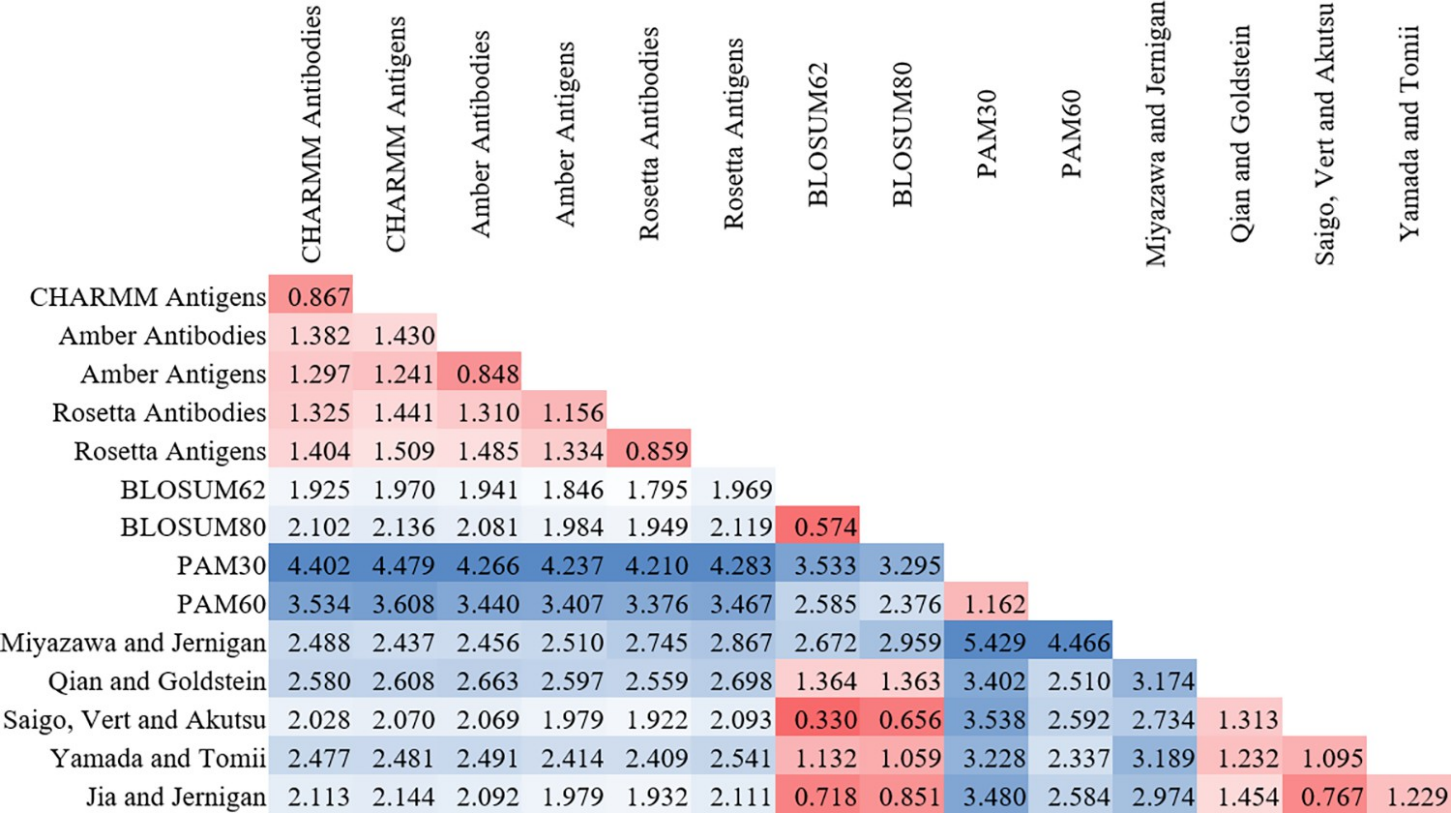
Error scores between similarity matrices. These values are a measure of how different equivalent mutations are in the different matrices. The values are shown using a heat map to make trends easier to visually identify, with smaller numbers in red and larger values in blue. The matrices calculated by the same force field (e.g. the CHARMM antibody and antigen matrices) have low scores. The error scores for the six matrices calculated in this work indicate similarity between the matrices, with a maximum value of 1.509. In contrast, the minimum error score of those matrices with any other matrix is 1.922.

The error scores calculated with Eq 5 are shown in Fig 2. The lowest error scores are between the set of the two BLOSUM matrices, the matrix of Saigo, Vert and Akutsu, and the matrix of Jia and Jernigan, suggesting those four matrices share common features. The error scores between PPI matrices calculated in this work by the same force field (e.g., the CHARMM antibody and antigen matrices) are also small compared to the other values in the matrices. The six matrices from this work have relatively low error scores compared to one another, with the maximum score between any of the six matrices being 1.509 between the antigen matrices of CHARMM and Rosetta. In contrast, the minimum score of any of those six matrices with any of the nine sequence similarity matrices is 1.922 between the Rosetta antibodies matrix and the matrix of Saigo, Vert and Akutsu. This indicates that while the six antibody-antigen interaction similarity matrices have differences between one another, they have more in common with one another than they do with previous matrices for protein sequence similarity.

## Discussion

The invention of reliable and accurate machine learning–based algorithms for protein structure prediction is transforming computational protein science and engineering. One of the key components of AlphaFold [1] and RoseTTAFold [2] is the extraction of information from similar protein sequences. The alignment of protein sequences is heavily dependent on similarity matrices, including PAM [32] and BLOSUM [33]. As machine learning method development shifts towards the prediction and design of PPIs, a possible strategy is to mimic what worked in AlphaFold and RoseTTAFold and include interaction similarity information.

This work described the calculation of antibody-protein interaction similarity matrices using three force fields, CHARMM [47], Amber [48], and Rosetta [49]. Each of these force fields has a long history of use in the fields of computational protein science and engineering and has been developed and optimized over the course of decades by hundreds of researchers. At the outset of the study, it was anticipated that the calculated matrices would exhibit similar properties but with force field dependent variations that would be relevant for different projects. Given that AlphaFold uses Amber as part of its final refinement of structures [1] while RosseTTAFold is integrated into the larger suite of Rosetta software, it seems likely that PPI methods will also utilize various force fields.

384 nonredundant complexes of antibodies binding to protein antigens were analyzed in this study. Analyzing antibody-antigen complexes was chosen for several reasons. First, antibodies are the archetypical binding protein. Due to their important and widespread therapeutic and experimental applications, they have been extensively studied in prior experimental and computational literature [50, 55–59]. Second, antibodies undergo an affinity maturation process to improve their binding to antigens [60] while the antigens remain unchanged. This provides an opportunity to compare how similarity matrices for PPIs differ for proteins that have mutated to improve the affinity of the interaction to those that have not.

The analyses only considered the hotspot residues in the proteins, defined as the seven residues in each antibody and antigen that contributed the most to the complexes’ binding affinities. Prior analyses have demonstrated that the most important residues contribute the significant majority of the binding energies in complexes, with the other residues in the interface making much smaller contributions [50]. The interfaces are much larger than the most important residues, with most having ~30 amino acids per protein. The choice was made to focus on only the most important residues to avoid biasing the data with binding energy changes from residues that were less important to the PPI. If these matrices prove to be of use, then future research can explore how they change when other force fields, other types of protein complexes, and additional residues are used in their calculation.

One feature observed while conducting this and prior work was that computationally predicted interaction energies often have significant deviations in magnitude from their experimental values. Further, we observed that the magnitude of the predicted energies increased with the buried surface area of the complexes. While the relative changes of energies for point mutations to complexes are informative, directly comparing the magnitudes was difficult. Therefore, Eq 2 was used to normalize the calculated values. Converting the energies into percentage changes facilitates the comparison of how important a given residue or mutation is between complexes.

The BLOSUM and PAM similarity matrices were the inspiration for this study, and therefore the matrices here were constructed to have similar features. In particular, the scores are all integer values, negative scores are detrimental while positive ones are beneficial, and the scores are logarithmically weighted. However, two key differences exist in the matrices. In PAM and BLOSUM, the scores for not mutating an amino acid are related to the frequency of that event in the protein sequences used to create the matrices. Here, all possible mutations were computationally calculated for each important residue so there was no corresponding natural score to use. Instead, the choice was made to have the sum of each row in the representative value matrices be equal to zero.

The other key difference is that the matrices calculated here are not symmetrical, because mutations can have different impacts on binding depending on their directionality. An example of this from Table 1 is that on average mutating glycine to arginine improved the predicted binding energy by 2.48% while mutating arginine to glycine worsened the predicted binding energy by 9.62%. This is to be expected: when arginine is important in a binding interface it is likely to be part of a salt bridge while glycine’s contributions are likely to come from its backbone. Mutating glycine to arginine at that position could still contribute to the backbone interactions while creating the potential for a salt bridge whereas mutating arginine to glycine is much more likely to remove a beneficial interaction. As these effects and magnitudes are not equal, similarity matrices for interface mutations should not be symmetrical.

Several features stand out in analyzing the similarity matrices in Tables 2–7. The most prominent are the consistent penalties for mutating charged and aromatic amino acids. When those residues are important to binding, mutating them resulted in the most negative scores with every force field for both antibodies and antigens. Interestingly, this even stood for mutations within the groups. Mutating an aromatic residue to another aromatic residue or a charged amino acid to the other amino acid with the same charge still had corresponding detrimental scores. This suggests that there are critical features in the interactions involving those residues that cannot easily be replaced even by similar chemical structures. Another commonality is that the scores for mutations between an antibody matrix and the antigen matrix calculated with the same force field were similar. For example, the scores for mutating charged residues were approximately -4 with Amber for both antibodies and antigens versus -3 with CHARMM and Rosetta.

While the CHARMM and Rosetta matrices showed somewhat less detrimental scores overall for the antigens, as was expected prior to calculation because the antigens had not been mutated to have improved binding with the antibodies, that was not the case with the Amber-calculated matrices. The overall trends in the matrices more closely matched the force field used to calculate them than the type of protein being mutated. In particular, CHARMM and Amber assigned more detrimental scores to mutations of polar amino acids than Rosetta did. In contrast, Rosetta had scores that were more punitive to mutating nonpolar residues and were more permissive of mutations to polar amino acids, especially serine.

To provide a more holistic assessment of the matrices compared to one another and to previous sequence similarity matrices, average difference scores and error scores were calculated. These values demonstrate that the matrices calculated by the same force field are very similar to one another. The difference scores between the CHARMM versus the Amber and Rosetta matrices were comparable to values with the sequence similarity matrices. However, the error calculations show that the six newly calculated matrices are more similar to one another than they are to any of the previously published sequence similarity matrices checked in this work. This suggests that there are effects of mutations in protein-protein interfaces that meaningfully differ compared to their effects on protein structures.

The authors do note that there are aspects of the antibody-protein similarity matrices that appear to reflect the historical priorities of the force fields used in this study. CHARMM and Amber share similar histories [47, 61], each beginning their development in the 1970s on code originally developed at Harvard for the purpose of *studying* macromolecules. Inherent in studying a protein is having a starting structure and how that structure interacts with its solvent and other molecules in its system. In contrast, the initial development of Rosetta occurred in the 1990s for the purpose of *predicting* protein structures [62], and the dominant force in protein folding is the hydrophobic effect [63]. The observation that Rosetta disfavors mutations to nonpolar residues more than Amber and CHARMM do is consistent with the intended purposes for which each program was created. Rosetta’s predictions that mutations of serine in antibody interfaces to phenylalanine, isoleucine, and valine are typically beneficial also contradict observed experimental trends in antibodies. In particular, antibodies have evolved so that there is an overabundance of serine in their binding surfaces [60]. It seems unlikely that mutations of a polar residue, which nature has chosen through evolution to be in antibody binding interfaces, to nonpolar residues should frequently be beneficial.

## Conclusions

To the authors’ knowledge, this work describes the first similarity matrices for PPIs, specifically for those in antibody-protein complexes. The calculated matrices have several interesting differences between them; however, the average difference and error scores calculated in Figs 1 and 2 demonstrate that the matrices have more in common with one another than they do with previous matrices for sequence similarity. This indicates that there are important differences in how mutations impact protein-protein interactions compared to how they affect protein structures. While it seems likely that the matrices developed in this work inherently reflect the features of the force fields used to calculate them, this should in no way be construed as a criticism of those force fields. They are excellent tools that were developed for specific purposes and have been continually improved upon for decades. They have each demonstrated innumerable times that they produce high-quality predictions of protein structures and properties. Rather, this should be interpreted as the observation that the statistical preferences inherent in their energy functions emerge when analyzing over 100,000 amino acid mutations. That the preferences would emerge was expected and was why three different force fields were used in this study. However, that the preferences would differ to the extent they do, especially for nonpolar amino acids and serine, was not anticipated. Those differences lead the authors to conclude that the similarity matrices created here can be used in force field specific applications, but further work is needed to identify a general PPI similarity matrix with consensus behaviors.

## Supporting information

S1 Table The representative values for mutations of antibody residues calculated by Amber. The broad trends are similar to those for CHARMM, with the key difference of mutating all amino acids is on average predicted to be detrimental.
(DOCX)

S2 Table The representative values for mutations of antibody residues calculated by Rosetta. As with Amber but unlike CHARMM, the representative values for not mutating every amino acid are positive.
(DOCX)

S3 Table The representative values for mutations of antigen residues calculated by CHARMM. Many of the trends are similar to those for the antibody residues calculated by CHARMM, although valine has a negative nonmutation score while glycine has a positive one.
(DOCX)

S4 Table The representative values for mutations of antigen residues calculated by Amber. The trends are qualitatively similar to those for the antibody mutations.
(DOCX)

S5 Table The representative values for mutations of antigen residues calculated by Rosetta. The strongest outliers in this data compared to the other tables are the number of favorable mutations for glycine and serine to nonpolar and aromatic residues.
(DOCX)

S1 File All calculated mutation energies. S1 File is a zip file containing every energy calculated in this analysis.
(ZIP)

## References

[1] Jumper J, Evans R, Pritzel A, Green T, Figurnov M, Ronneberger O, et al. Highly accurate protein structure prediction with AlphaFold. Nature. 2021;596(7873):583–9. doi: 10.1038/s41586-021-03819-2 34265844 PMC8371605

[2] Baek M, DiMaio F, Anishchenko I, Dauparas J, Ovchinnikov S, Lee GR, et al. Accurate prediction of protein structures and interactions using a three-track neural network. Science. 2021;373(6557):871–6. doi: 10.1126/science.abj8754 34282049 PMC7612213

[3] Chakravarty D, Porter LL. AlphaFold2 fails to predict protein fold switching. Protein Science. 2022;31(6):e4353. doi: 10.1002/pro.4353 35634782 PMC9134877

[4] Pak MA, Markhieva KA, Novikova MS, Petrov DS, Vorobyev IS, Maksimova ES, et al. Using AlphaFold to predict the impact of single mutations on protein stability and function. PLOS ONE. 2023;18(3):e0282689. doi: 10.1371/journal.pone.0282689 36928239 PMC10019719

[5] Outeiral C, Nissley DA, Deane CM. Current structure predictors are not learning the physics of protein folding. Bioinformatics. 2022;38(7):1881–7. doi: 10.1093/bioinformatics/btab881 35099504 PMC8963306

[6] Nooren IMA, Thornton JM. Diversity of protein–protein interactions. The EMBO Journal. 2003;22(14):3486–92. doi: 10.1093/emboj/cdg359 12853464 PMC165629

[7] Acuner Ozbabacan SE, Engin HB, Gursoy A, Keskin O. Transient protein–protein interactions. Protein Engineering, Design and Selection. 2011;24(9):635–48. doi: 10.1093/protein/gzr025 21676899

[8] Blundell TL, Burke DF, Chirgadze D, Dhanaraj V, Hyvonen M, Axel Innis C, et al. Protein-protein interactions in receptor activation and intracellular signalling. Biological Chemistry. 2000;381(9–10):955–9. doi: 10.1515/BC.2000.117 11076027

[9] Valencia A, Pazos F. Computational methods for the prediction of protein interactions. Current Opinion in Structural Biology. 2002;12(3):368–73. doi: 10.1016/s0959-440x(02)00333-0 12127457

[10] Glaser F, Steinberg DM, Vakser IA, Ben-Tal N. Residue frequencies and pairing preferences at protein–protein interfaces. Proteins: Structure, Function, and Bioinformatics. 2001;43(2):89–102. doi: 10.1002/1097-0134(20010501)43:2&lt;89::AID-PROT1021&gt;3.0.CO;2-H 11276079

[11] Lo Conte L, Chothia C, Janin J. The atomic structure of protein–protein recognition sites. J Mol Biol. 1999;285:2177–98-–98. doi: 10.1006/jmbi.1998.2439 9925793

[12] Bordner AJ, Abagyan R. Statistical analysis and prediction of protein–protein interfaces. Proteins: Structure, Function, and Bioinformatics. 2005;60(3):353–66. doi: 10.1002/prot.20433 15906321

[13] Moreira IS, Fernandes PA, Ramos MJ. Hot spots—A review of the protein–protein interface determinant amino-acid residues. Proteins: Structure, Function, and Bioinformatics. 2007;68(4):803–12. doi: 10.1002/prot.21396 17546660

[14] Russell RB, Alber F, Aloy P, Davis FP, Korkin D, Pichaud M, et al. A structural perspective on protein–protein interactions. Current Opinion in Structural Biology. 2004;14(3):313–24. doi: 10.1016/j.sbi.2004.04.006 15193311

[15] Verkhivker GM, Bouzida D, Gehlhaar DK, Rejto PA, Freer ST, Rose PW. Computational detection of the binding-site hot spot at the remodeled human growth hormone–receptor interface. Proteins: Structure, Function, and Bioinformatics. 2003;53(2):201–19. doi: 10.1002/prot.10456 14517972

[16] Kortemme T, Baker D. Computational design of protein–protein interactions. Current Opinion in Chemical Biology. 2004;8(1):91–7. doi: 10.1016/j.cbpa.2003.12.008 15036162

[17] Ramaraj T, Angel T, Dratz EA, Jesaitis AJ, Mumey B. Antigen-antibody interface properties: Composition, residue interactions, and features of 53 non-redundant structures. Biochimica et Biophysica Acta—Proteins and Proteomics. 2012;1824(3):520–32. doi: 10.1016/j.bbapap.2011.12.007 22246133 PMC3443979

[18] Hu J, Li J, Chen N, Zhang X. Conservation of hot regions in protein–protein interaction in evolution. Methods. 2016;110(4):73–80. doi: 10.1016/j.ymeth.2016.06.020 27346249

[19] Yan C, Wu F, Jernigan RL, Dobbs D, Honavar V. Characterization of Protein–Protein Interfaces. The Protein Journal. 2008;27(1):59–70. doi: 10.1007/s10930-007-9108-x 17851740 PMC2566606

[20] Tokuriki N, Tawfik DS. Protein Dynamism and Evolvability. Science. 2009;324(5924):203 LP-7. doi: 10.1126/science.1169375 19359577

[21] Jubb HC, Pandurangan AP, Turner MA, Ochoa-Montaño B, Blundell TL, Ascher DB. Mutations at protein-protein interfaces: Small changes over big surfaces have large impacts on human health. Progress in Biophysics and Molecular Biology. 2017;128:3–13. doi: 10.1016/j.pbiomolbio.2016.10.002 27913149

[22] Moal IH, Fernández-Recio J. SKEMPI: a Structural Kinetic and Energetic database of Mutant Protein Interactions and its use in empirical models. Bioinformatics. 2012;28(20):2600–7. doi: 10.1093/bioinformatics/bts489 22859501

[23] Jankauskaite J, Jiménez-García B, Dapkunas J, Fernández-Recio J, Moal IH. SKEMPI 2.0: an updated benchmark of changes in protein-protein binding energy, kinetics and thermodynamics upon mutation. Bioinformatics (Oxford, England). 2019;35(3):462–9. doi: 10.1093/bioinformatics/bty635 30020414 PMC6361233

[24] Moretti R, Fleishman SJ, Agius R, Torchala M, Bates PA, Kastritis PL, et al. Community-wide evaluation of methods for predicting the effect of mutations on protein–protein interactions. Proteins: Structure, Function, and Bioinformatics. 2013;81(11):1980–7. doi: 10.1002/prot.24356 23843247 PMC4143140

[25] Parthiban V, Gromiha MM, Schomburg D. CUPSAT: prediction of protein stability upon point mutations. Nucleic Acids Research. 2006;34(suppl_2):W239–W42. doi: 10.1093/nar/gkl190 16845001 PMC1538884

[26] Tokuriki N, Stricher F, Schymkowitz J, Serrano L, Tawfik DS. The Stability Effects of Protein Mutations Appear to be Universally Distributed. Journal of Molecular Biology. 2007;369(5):1318–32. doi: 10.1016/j.jmb.2007.03.069 17482644

[27] Berliner N, Teyra J, Çolak R, Garcia Lopez S, Kim PM. Combining Structural Modeling with Ensemble Machine Learning to Accurately Predict Protein Fold Stability and Binding Affinity Effects upon Mutation. PLOS ONE. 2014;9(9):e107353–e. doi: 10.1371/journal.pone.0107353 25243403 PMC4170975

[28] Pires DEV, Ascher DB, Blundell TL. mCSM: predicting the effects of mutations in proteins using graph-based signatures. Bioinformatics. 2013;30(3):335–42. doi: 10.1093/bioinformatics/btt691 24281696 PMC3904523

[29] Dehouck Y, Kwasigroch JM, Rooman M, Gilis D. BeAtMuSiC: prediction of changes in protein–protein binding affinity on mutations. Nucleic Acids Research. 2013;41(W1):W333–W9. doi: 10.1093/nar/gkt450 23723246 PMC3692068

[30] Simões ICM, Costa IPD, Coimbra JTS, Ramos MJ, Fernandes PA. New Parameters for Higher Accuracy in the Computation of Binding Free Energy Differences upon Alanine Scanning Mutagenesis on Protein–Protein Interfaces. Journal of Chemical Information and Modeling. 2017;57(1):60–72. doi: 10.1021/acs.jcim.6b00378 27936711

[31] Li M, Petukh M, Alexov E, Panchenko AR. Predicting the Impact of Missense Mutations on Protein–Protein Binding Affinity. Journal of Chemical Theory and Computation. 2014;10(4):1770–80. doi: 10.1021/ct401022c 24803870 PMC3985714

[32] Dayhoff MO SR, Orcutt BC. A model of Evolutionary Change in Proteins. Washington, DC: National Biomedical Research Foundation; 1978.

[33] Henikoff S, Henikoff JG. Amino acid substitution matrices from protein blocks. Proceedings of the National Academy of Sciences. 1992;89(22):10915 LP-9. doi: 10.1073/pnas.89.22.10915 1438297 PMC50453

[34] Miyazawa S, Jernigan RL. A new substitution matrix for protein sequence searches based on contact frequencies in protein structures. Protein Engineering, Design and Selection. 1993;6(3):267–78. doi: 10.1093/protein/6.3.267 8506261

[35] Müller T, Spang R, Vingron M. Estimating Amino Acid Substitution Models: A Comparison of Dayhoff’s Estimator, the Resolvent Approach and a Maximum Likelihood Method. Molecular Biology and Evolution. 2002;19(1):8–13. doi: 10.1093/oxfordjournals.molbev.a003985 11752185

[36] Vilim RB, Cunningham RM, Lu B, Kheradpour P, Stevens FJ. Fold-specific substitution matrices for protein classification. Bioinformatics. 2004;20(6):847–53. doi: 10.1093/bioinformatics/btg492 14764567

[37] Jimenez-Morales D, Adamian L, Liang J, editors. Detecting remote homologues using scoring matrices calculated from the estimation of amino acid substitution rates of beta-barrel membrane proteins. 2008 30th Annual International Conference of the IEEE Engineering in Medicine and Biology Society; 2008 20–25 Aug. 2008. 10.1109/IEMBS.2008.4649414 PMC2630510 19162917

[38] Tomii K, Yamada K. Systematic Exploration of an Efficient Amino Acid Substitution Matrix: MIQS. In: Carugo O, Eisenhaber F, editors. Data Mining Techniques for the Life Sciences. New York, NY: Springer New York; 2016. p. 211–23. 10.1007/978-1-4939-3572-7_11 27115635

[39] Jung J, Lee B. Use of residue pairs in protein sequence-sequence and sequence-structure alignments. Protein Science. 2000;9(8):1576–88. doi: 10.1110/ps.9.8.1576 10975579 PMC2144723

[40] Edgar RC. Optimizing substitution matrix choice and gap parameters for sequence alignment. BMC Bioinformatics. 2009;10(1):396. doi: 10.1186/1471-2105-10-396 19954534 PMC2791778

[41] Qian B, Goldstein RA. Optimization of a new score function for the generation of accurate alignments. Proteins: Structure, Function, and Bioinformatics. 2002;48(4):605–10. doi: 10.1002/prot.10132 12211027

[42] Saigo H, Vert J-P, Akutsu T. Optimizing amino acid substitution matrices with a local alignment kernel. BMC Bioinformatics. 2006;7(1):246. doi: 10.1186/1471-2105-7-246 16677385 PMC1513605

[43] Yamada K, Tomii K. Revisiting amino acid substitution matrices for identifying distantly related proteins. Bioinformatics. 2014;30(3):317–25. doi: 10.1093/bioinformatics/btt694 24281694 PMC3904525

[44] Song D, Chen J, Chen G, Li N, Li J, Fan J, et al. Parameterized BLOSUM Matrices for Protein Alignment. IEEE/ACM Transactions on Computational Biology and Bioinformatics. 2015;12(3):686–94. doi: 10.1109/TCBB.2014.2366126 26357279

[45] Jia K, Jernigan RL. New amino acid substitution matrix brings sequence alignments into agreement with structure matches. Proteins: Structure, Function, and Bioinformatics. 2021;89(6):671–82. doi: 10.1002/prot.26050 33469973 PMC8641535

[46] Altschul SF, Gish W, Miller W, Myers EW, Lipman DJ. Basic local alignment search tool. Journal of Molecular Biology. 1990;215(3):403–10. doi: 10.1016/S0022-2836(05)80360-2 2231712

[47] Brooks BR, Brooks 3rd CL, Mackerell AD Jr, Nilsson L, Petrella RJ, Roux B, et al. CHARMM: the biomolecular simulation program. Journal of computational chemistry. 2009;30(10):1545–614. doi: 10.1002/jcc.21287 19444816 PMC2810661

[48] Case DA, Cerutti DS, Cheatham Iii TE, Darden TA, Duke RE, Giese TJ, et al. AMBER 2017. University of California, San Francisco 2017.

[49] Alford RF, Leaver-Fay A, Jeliazkov JR, O’Meara MJ, DiMaio FP, Park H, et al. The Rosetta All-Atom Energy Function for Macromolecular Modeling and Design. Journal of Chemical Theory and Computation. 2017;13(6):3031–48. doi: 10.1021/acs.jctc.7b00125 28430426 PMC5717763

[50] Chauhan VM, Islam S, Vroom A, Pantazes R. Development and Analyses of a Database of Antibody–Antigen Complexes. Computer Aided Chemical Engineering. 2018;44:2113–8. doi: 10.1016/B978-0-444-64241-7.50347–5

[51] Haberthür U, Caflisch A. FACTS: Fast analytical continuum treatment of solvation. Journal of Computational Chemistry. 2008;29(5):701–15. doi: 10.1002/jcc.20832 17918282

[52] Maier JA, Martinez C, Kasavajhala K, Wickstrom L, Hauser KE, Simmerling C. ff14SB: Improving the Accuracy of Protein Side Chain and Backbone Parameters from ff99SB. Journal of Chemical Theory and Computation. 2015;11(8):3696–713. doi: 10.1021/acs.jctc.5b00255 26574453 PMC4821407

[53] Dunbrack RL Jr, Cohen FE. Bayesian statistical analysis of protein side-chain rotamer preferences. Protein Science. 1997;6(8):1661–81. doi: 10.1002/pro.5560060807 9260279 PMC2143774

[54] Whitley E BJ. Statistics review 1: presenting and summarising data. Critical Care. 2002;6(1):66–71. Epub 2001 Nov 29. doi: 10.1186/cc1455 PubMed Central PMCID: PMC137399. 11940268 PMC137399

[55] Chen S-wW, Pellequer MHVVR, Jean L. Structure-Activity Relationships in Peptide-Antibody Complexes: Implications for Epitope Prediction and Development of Synthetic Peptide Vaccines. Current Medicinal Chemistry 2009. p. 953–64. doi: 10.2174/092986709787581914 19275605

[56] Nguyen MN, Pradhan MR, Verma C, Zhong P. The interfacial character of antibody paratopes: Analysis of antibody-antigen structures. Bioinformatics. 2017;33(19):2971–6. doi: 10.1093/bioinformatics/btx389 28633399

[57] Robin G, Sato Y, Desplancq D, Rochel N, Weiss E, Martineau P. Restricted Diversity of Antigen Binding Residues of Antibodies Revealed by Computational Alanine Scanning of 227 Antibody–Antigen Complexes. Journal of Molecular Biology. 2014;426(22):3729–43. doi: 10.1016/j.jmb.2014.08.013 25174334

[58] Kuroda D, Gray JJ. Shape complementarity and hydrogen bond preferences in protein-protein interfaces: Implications for antibody modeling and protein-protein docking. Bioinformatics. 2016;32(16):2451–6. doi: 10.1093/bioinformatics/btw197 27153634 PMC4978935

[59] Fleishman SJ, Khare SD, Koga N, Baker D. Restricted sidechain plasticity in the structures of native proteins and complexes. Protein Science. 2011;20(4):753–7. doi: 10.1002/pro.604 21432939 PMC3081553

[60] Burkovitz A, Sela-Culang I, Ofran Y. Large-scale analysis of somatic hypermutations in antibodies reveals which structural regions, positions and amino acids are modified to improve affinity. The FEBS Journal. 2014;281(1):306–19. doi: 10.1111/febs.12597 24279419

[61] Pearlman DA, Case DA, Caldwell JW, Ross WS, Cheatham TE, DeBolt S, et al. AMBER, a package of computer programs for applying molecular mechanics, normal mode analysis, molecular dynamics and free energy calculations to simulate the structural and energetic properties of molecules. Computer Physics Communications. 1995;91(1):1–41. 10.1016/0010-4655(95)00041-D.

[62] Simons KT, Kooperberg C, Huang E, Baker D. Assembly of protein tertiary structures from fragments with similar local sequences using simulated annealing and bayesian scoring functions11Edited by F. E. Cohen. Journal of Molecular Biology. 1997;268(1):209–25. 10.1006/jmbi.1997.0959. 9149153

[63] Dill KA. Dominant forces in protein folding. Biochemistry. 1990;29(31):7133–55. doi: 10.1021/bi00483a001 2207096

