# Supplementary material for: Developing similarity matrices for antibody-protein binding interactions
Source: PLoS One. 2023 Oct 26;18(10):e0293606. doi: 10.1371/journal.pone.0293606 (PMC10602319; doi:10.1371/journal.pone.0293606)
Supplement: S1 Table — The broad trends are similar to those for CHARMM, with the key difference of mutating all amino acids is on average predicted to be detrimental. (DOCX) [file pone.0293606.s001.docx]

**Supplemental Table 1: The representative values for mutations of antibody residues calculated by Amber.** The broad trends are similar to those for CHARMM, with the key difference of mutating all amino acids is on average predicted to be detrimental.

|  | A | C | D | E | F | G | H | I | K | L | M | N | P | Q | R | S | T | V | W | Y |
| --- | --- | --- | --- | --- | --- | --- | --- | --- | --- | --- | --- | --- | --- | --- | --- | --- | --- | --- | --- | --- |
| A | 60.76 | -1.51 | -6.48 | -3.89 | -2.15 | -4.09 | -3.07 | -3.62 | -1.03 | -3.63 | -4.71 | -2.68 | -4.69 | -4.39 | 4.12 | -4.41 | -1.10 | -2.47 | -3.39 | -7.58 |
| C | -5.04 | 116.05 | -8.14 | -5.24 | -8.75 | -4.39 | -4.16 | -6.83 | -7.99 | -4.86 | -7.11 | -5.03 | -5.00 | -6.63 | -3.17 | -4.27 | -5.59 | -4.40 | -7.33 | -12.15 |
| D | -12.99 | -12.71 | 234.98 | -7.72 | -12.81 | -12.63 | -12.46 | -12.59 | -13.30 | -12.99 | -12.35 | -11.31 | -13.10 | -11.44 | -13.09 | -12.76 | -12.32 | -13.02 | -12.39 | -13.01 |
| E | -12.99 | -12.04 | -8.62 | 229.74 | -12.39 | -13.80 | -12.38 | -11.60 | -12.73 | -12.49 | -12.79 | -11.02 | -12.90 | -10.48 | -12.19 | -12.71 | -12.48 | -12.83 | -11.92 | -11.40 |
| F | -6.26 | -6.42 | -6.08 | -4.52 | 110.98 | -7.07 | -4.75 | -5.65 | -5.54 | -5.98 | -5.50 | -5.87 | -7.19 | -5.15 | -5.87 | -6.19 | -6.01 | -6.02 | -6.04 | -4.90 |
| G | -3.23 | -5.04 | -4.64 | 0.09 | -3.91 | 60.40 | -2.82 | -3.42 | -1.59 | -5.34 | -3.61 | -2.52 | -7.84 | -1.61 | 1.05 | -1.98 | -4.00 | -5.74 | -1.02 | -3.25 |
| H | -6.54 | -7.63 | -1.11 | -3.48 | -4.20 | -8.78 | 88.28 | -6.78 | 0.96 | -3.76 | -6.03 | -2.01 | -8.39 | -0.03 | -1.81 | -5.17 | -6.68 | -6.05 | -4.11 | -6.67 |
| I | -3.06 | -3.81 | 1.33 | 1.99 | -1.08 | -3.72 | -1.01 | 24.34 | 0.48 | -2.95 | -2.70 | -1.60 | -3.97 | -0.40 | 2.46 | -1.69 | -1.55 | -2.26 | -0.40 | -0.42 |
| K | -13.04 | -12.31 | -13.60 | -13.45 | -12.87 | -12.12 | -12.39 | -12.87 | 236.82 | -12.25 | -13.01 | -12.14 | -12.39 | -12.22 | -9.43 | -12.63 | -12.55 | -12.41 | -13.03 | -12.11 |
| L | -5.64 | -3.99 | -4.65 | -2.04 | -2.98 | -5.94 | -2.76 | -3.59 | 0.94 | 61.56 | -3.24 | -2.56 | -4.65 | -2.97 | -2.44 | -4.16 | -2.40 | -3.22 | -1.95 | -3.33 |
| M | -6.64 | -4.66 | -4.09 | -2.21 | -3.31 | -6.57 | -2.96 | -2.80 | -4.02 | -3.25 | 72.09 | -4.75 | -2.85 | -5.23 | -1.99 | -3.64 | -4.97 | -3.11 | -1.81 | -3.23 |
| N | -9.17 | -8.24 | -7.55 | -8.15 | -8.21 | -9.29 | -8.17 | -8.17 | -5.10 | -8.01 | -7.72 | 146.66 | -7.84 | -6.73 | -6.73 | -6.90 | -7.29 | -8.41 | -7.74 | -7.27 |
| P | -3.68 | -4.05 | -3.45 | -4.29 | -8.08 | -2.46 | -12.11 | -5.76 | -1.48 | -4.41 | -8.62 | -5.53 | 91.87 | 2.01 | -5.19 | -3.77 | -3.78 | -5.18 | -4.98 | -7.05 |
| Q | -5.95 | -5.35 | -5.86 | -5.38 | -4.29 | -7.26 | -5.36 | -4.63 | -3.55 | -6.27 | -5.25 | -5.05 | -5.89 | 101.70 | -4.26 | -5.74 | -5.94 | -5.93 | -4.12 | -5.64 |
| R | -14.96 | -14.02 | -15.31 | -14.47 | -13.95 | -15.28 | -14.07 | -14.43 | -11.58 | -14.71 | -14.47 | -14.00 | -14.47 | -13.86 | 271.74 | -14.67 | -14.64 | -14.79 | -13.83 | -14.23 |
| S | -6.72 | -5.43 | -5.30 | -5.46 | -7.05 | -7.02 | -6.73 | -6.99 | -4.96 | -6.82 | -6.87 | -4.09 | -8.01 | -5.20 | -4.12 | 115.90 | -4.12 | -6.87 | -6.67 | -7.48 |
| T | -6.84 | -5.83 | -4.26 | -7.14 | -7.77 | -7.54 | -6.94 | -7.30 | -4.53 | -7.04 | -6.55 | -5.31 | -6.37 | -5.08 | -3.19 | -4.36 | 115.41 | -6.11 | -6.57 | -6.69 |
| V | -5.17 | -3.01 | 0.45 | -3.74 | -3.53 | -7.27 | -3.86 | -3.07 | 0.95 | -4.78 | -2.35 | -1.08 | -3.69 | -1.79 | -1.41 | -3.55 | -3.90 | 57.04 | -2.12 | -4.12 |
| W | -9.73 | -9.19 | -9.15 | -8.95 | -8.10 | -9.30 | -7.96 | -8.57 | -7.55 | -9.07 | -8.35 | -8.96 | -10.16 | -7.72 | -7.37 | -8.93 | -8.03 | -9.56 | 164.92 | -8.27 |
| Y | -9.13 | -8.42 | -8.28 | -7.76 | -7.71 | -9.61 | -7.48 | -8.31 | -7.19 | -8.45 | -8.18 | -7.77 | -8.84 | -7.83 | -5.99 | -8.42 | -8.36 | -8.61 | -7.68 | 154.02 |
