# Supplementary material for: Developing similarity matrices for antibody-protein binding interactions
Source: PLoS One. 2023 Oct 26;18(10):e0293606. doi: 10.1371/journal.pone.0293606 (PMC10602319; doi:10.1371/journal.pone.0293606)
Supplement: S2 Table — As with Amber but unlike CHARMM, the representative values for not mutating every amino acid are positive. (DOCX) [file pone.0293606.s002.docx]

**Supplemental Table 2: The representative values for mutations of antibody residues calculated by Rosetta.** As with Amber but unlike CHARMM, the representative values for not mutating every amino acid are positive.

|  | A | C | D | E | F | G | H | I | K | L | M | N | P | Q | R | S | T | V | W | Y |
| --- | --- | --- | --- | --- | --- | --- | --- | --- | --- | --- | --- | --- | --- | --- | --- | --- | --- | --- | --- | --- |
| A | 46.77 | 1.34 | -6.26 | -2.34 | 0.42 | -2.97 | -3.67 | 0.56 | -4.17 | -1.16 | -1.81 | -4.30 | -4.08 | -2.76 | -5.10 | -4.46 | -0.18 | 0.44 | -1.29 | -4.98 |
| C | -7.02 | 198.72 | -10.13 | -7.91 | -8.49 | -7.73 | -14.48 | -12.86 | -14.66 | -12.94 | -5.08 | -11.11 | -11.50 | -8.17 | -15.15 | -11.71 | -10.70 | -8.24 | -9.35 | -11.50 |
| D | -8.54 | -9.37 | 168.39 | -6.05 | -7.68 | -9.27 | -9.66 | -8.49 | -11.28 | -8.18 | -8.86 | -9.16 | -9.66 | -7.59 | -11.30 | -9.34 | -9.04 | -8.36 | -8.22 | -8.35 |
| E | -8.80 | -7.60 | -5.50 | 157.68 | -6.92 | -9.69 | -9.41 | -8.48 | -11.47 | -7.82 | -7.44 | -7.10 | -9.61 | -7.13 | -10.09 | -9.12 | -8.99 | -7.29 | -8.32 | -6.92 |
| F | -6.29 | -7.19 | -9.21 | -8.47 | 141.45 | -9.09 | -7.36 | -5.02 | -7.95 | -5.69 | -6.05 | -8.45 | -9.19 | -8.25 | -8.72 | -9.02 | -7.43 | -6.32 | -6.24 | -5.51 |
| G | -1.48 | -1.15 | -3.98 | -4.37 | -1.09 | 52.32 | -3.07 | -2.24 | -4.60 | -1.48 | -1.17 | -2.62 | -7.87 | -2.37 | -3.55 | -4.09 | -4.82 | -1.78 | 0.66 | -1.26 |
| H | -2.78 | -3.75 | -5.19 | -7.31 | -1.82 | -4.62 | 71.69 | -1.63 | -4.64 | -2.16 | -2.67 | -2.79 | -4.49 | -3.12 | -5.49 | -3.96 | -2.73 | -5.37 | -3.80 | -3.38 |
| I | -7.03 | -5.34 | -10.30 | -9.67 | -4.21 | -6.83 | -8.36 | 147.84 | -9.30 | -5.50 | -5.28 | -9.25 | -9.11 | -7.80 | -10.35 | -9.76 | -9.18 | -5.63 | -6.92 | -8.02 |
| K | -9.32 | -8.13 | -8.05 | -9.70 | -7.39 | -9.77 | -9.78 | -7.01 | 155.84 | -6.53 | -6.51 | -8.01 | -9.22 | -7.22 | -8.15 | -10.49 | -8.23 | -7.90 | -6.79 | -7.66 |
| L | -4.90 | -4.13 | -7.64 | -8.46 | -2.01 | -7.03 | -4.84 | -3.82 | -7.90 | 113.77 | -5.45 | -7.01 | -8.44 | -5.43 | -7.32 | -7.92 | -6.87 | -4.46 | -5.95 | -4.18 |
| M | -8.78 | -8.26 | -11.71 | -9.58 | -5.40 | -9.11 | -7.28 | -4.77 | -7.72 | -3.67 | 138.26 | -8.23 | -8.03 | -9.11 | -8.47 | -8.63 | -6.52 | -3.32 | -1.80 | -7.89 |
| N | -4.92 | -4.69 | -5.50 | -5.44 | -6.20 | -5.18 | -7.29 | -6.13 | -7.94 | -4.56 | -3.16 | 109.12 | -6.70 | -4.51 | -7.93 | -8.01 | -5.99 | -5.70 | -3.43 | -5.85 |
| P | -2.99 | 0.82 | -11.78 | -5.51 | -2.93 | -4.38 | -11.85 | -4.86 | -8.68 | 0.78 | -1.39 | -3.04 | 71.47 | -9.00 | -2.07 | -3.52 | -0.32 | -1.37 | 3.26 | -2.64 |
| Q | -5.03 | -6.99 | -7.85 | -6.18 | -3.52 | -8.42 | -5.35 | -5.78 | -7.41 | -4.69 | -5.03 | -5.51 | -7.83 | 111.86 | -3.71 | -6.97 | -7.34 | -5.41 | -4.64 | -4.19 |
| R | -8.45 | -8.13 | -9.28 | -10.09 | -7.12 | -8.46 | -8.47 | -6.90 | -7.16 | -8.67 | -6.42 | -8.60 | -9.34 | -7.69 | 155.82 | -8.72 | -9.11 | -7.80 | -7.77 | -7.64 |
| S | -0.18 | -1.35 | -3.49 | -3.84 | 1.08 | -1.71 | -2.43 | 0.73 | -1.99 | -0.72 | -1.34 | -1.61 | -2.20 | -1.18 | -2.88 | 27.02 | -1.98 | 0.44 | -1.86 | -0.54 |
| T | -4.71 | -2.74 | -5.54 | -6.46 | -2.21 | -5.09 | -6.25 | -2.57 | -4.45 | -1.70 | -2.34 | -5.77 | -6.05 | -3.81 | -5.66 | -5.19 | 83.85 | -3.52 | -4.90 | -4.89 |
| V | -4.42 | -4.79 | -5.52 | -7.22 | -3.73 | -3.88 | -6.95 | -2.40 | -9.51 | -4.21 | -3.25 | -6.85 | -6.72 | -4.57 | -6.70 | -6.93 | -4.82 | 106.19 | -5.46 | -8.28 |
| W | -10.83 | -9.50 | -11.23 | -11.29 | -8.69 | -10.92 | -11.08 | -9.09 | -12.57 | -9.43 | -9.10 | -10.84 | -10.47 | -10.89 | -11.15 | -11.64 | -10.20 | -8.76 | 197.27 | -9.58 |
| Y | -7.44 | -6.78 | -9.38 | -9.41 | -5.33 | -8.43 | -8.14 | -5.68 | -9.15 | -5.98 | -6.15 | -8.31 | -8.68 | -7.70 | -7.90 | -9.00 | -7.79 | -5.94 | -7.24 | 144.39 |
