# Supplementary material for: Developing similarity matrices for antibody-protein binding interactions
Source: PLoS One. 2023 Oct 26;18(10):e0293606. doi: 10.1371/journal.pone.0293606 (PMC10602319; doi:10.1371/journal.pone.0293606)
Supplement: S3 Table — Many of the trends are similar to those for the antibody residues calculated by CHARMM, although valine has a negative nonmutation score while glycine has a positive one. (DOCX) [file pone.0293606.s003.docx]

**Supplemental Table 3: The representative values for mutations of antigen residues calculated by CHARMM.** Many of the trends are similar to those for the antibody residues calculated by CHARMM, although valine has a negative nonmutation score while glycine has a positive one.

|  | A | C | D | E | F | G | H | I | K | L | M | N | P | Q | R | S | T | V | W | Y |
| --- | --- | --- | --- | --- | --- | --- | --- | --- | --- | --- | --- | --- | --- | --- | --- | --- | --- | --- | --- | --- |
| A | -7.10 | 1.47 | -7.32 | -2.15 | 1.29 | -0.88 | 1.57 | 0.74 | -0.72 | 1.42 | 1.28 | 0.69 | -0.40 | 1.36 | 2.16 | 0.15 | 0.37 | 0.65 | 2.89 | 2.53 |
| C | -0.39 | 35.02 | -4.24 | -6.26 | -2.09 | -1.14 | -1.18 | -1.59 | -3.80 | -2.19 | -0.07 | -1.34 | -1.49 | -0.51 | -3.49 | -0.78 | -1.05 | -1.79 | -0.35 | -1.28 |
| D | -6.74 | -5.69 | 108.75 | -2.53 | -5.39 | -6.79 | -5.55 | -5.91 | -7.36 | -6.17 | -5.39 | -5.68 | -6.14 | -4.91 | -6.46 | -6.10 | -5.76 | -5.89 | -5.07 | -5.23 |
| E | -7.75 | -7.31 | -4.69 | 131.80 | -6.87 | -7.94 | -6.17 | -6.92 | -8.20 | -7.04 | -6.76 | -6.39 | -7.57 | -6.11 | -6.98 | -7.67 | -7.42 | -7.28 | -6.42 | -6.30 |
| F | -5.51 | -3.45 | -9.54 | -10.08 | 86.14 | -5.44 | -3.48 | -2.40 | -6.57 | -2.81 | -2.01 | -4.45 | -5.85 | -3.97 | -4.64 | -5.17 | -4.00 | -3.77 | -2.63 | -0.36 |
| G | -0.23 | 1.36 | -4.14 | -3.40 | 0.49 | 5.86 | -0.54 | -0.63 | -1.51 | 0.56 | 0.77 | -0.12 | -0.71 | 0.62 | 1.93 | -0.20 | 0.02 | -0.56 | 0.22 | 0.20 |
| H | -3.43 | -1.62 | -6.48 | -5.36 | -1.64 | -4.27 | 56.92 | -2.87 | -3.20 | -2.64 | -1.31 | -3.04 | -2.67 | -1.82 | -2.08 | -3.68 | -3.18 | -3.33 | -2.76 | -1.55 |
| I | -3.41 | -1.49 | -6.30 | -11.08 | -0.55 | -4.67 | -2.34 | 47.22 | -3.51 | -1.25 | -0.30 | -2.20 | -2.57 | 0.62 | -0.06 | -2.51 | -2.18 | -1.24 | -0.91 | -1.29 |
| K | -5.73 | -4.88 | -8.62 | -8.01 | -5.51 | -5.87 | -5.36 | -5.23 | 104.41 | -5.55 | -5.13 | -5.45 | -5.58 | -4.95 | -1.39 | -5.75 | -5.72 | -5.80 | -4.98 | -4.93 |
| L | -2.35 | -1.34 | -7.46 | -4.88 | -0.29 | -3.33 | -1.05 | -1.10 | -2.19 | 37.03 | -0.04 | -2.18 | -2.37 | -0.91 | -0.96 | -2.36 | -1.77 | -1.39 | -0.21 | -0.86 |
| M | -3.97 | -1.99 | -6.50 | -3.14 | -1.99 | -5.40 | -3.10 | -2.49 | -0.74 | -2.67 | 60.68 | -4.71 | -4.01 | -4.55 | -0.87 | -4.44 | -4.14 | -3.77 | -1.13 | -1.08 |
| N | -4.33 | -2.19 | -5.76 | -5.58 | -2.20 | -4.83 | -2.71 | -2.90 | -3.76 | -2.93 | -2.31 | 60.15 | -3.77 | -1.87 | -1.58 | -3.09 | -3.19 | -3.36 | -1.57 | -2.25 |
| P | -1.41 | -0.92 | -2.53 | -2.24 | -1.02 | -2.13 | -0.98 | -0.60 | -0.99 | -0.88 | 0.24 | -1.28 | 15.20 | -0.29 | 0.83 | -1.77 | -0.89 | -0.64 | 2.09 | 0.20 |
| Q | -5.10 | -4.10 | -6.94 | -5.84 | -3.40 | -5.57 | -2.80 | -4.00 | -5.00 | -3.81 | -3.18 | -3.84 | -4.95 | 82.26 | -3.51 | -4.81 | -4.51 | -4.44 | -3.30 | -3.19 |
| R | -9.44 | -8.68 | -11.46 | -11.47 | -8.42 | -9.58 | -8.78 | -8.56 | -8.21 | -8.87 | -8.08 | -8.76 | -9.05 | -8.48 | 171.24 | -9.33 | -9.28 | -8.85 | -8.13 | -7.84 |
| S | -3.56 | -1.66 | -6.16 | -4.41 | -2.56 | -4.74 | -1.49 | -2.71 | -4.24 | -3.86 | -1.85 | -1.65 | -3.30 | -1.57 | -0.70 | 52.59 | -0.86 | -3.32 | -1.67 | -2.28 |
| T | -4.10 | -0.97 | -7.88 | -5.49 | -1.27 | -5.17 | -1.93 | -3.29 | -4.23 | -3.30 | -1.76 | -1.91 | -3.38 | -1.59 | -1.08 | -1.25 | 53.58 | -3.19 | -1.01 | -0.81 |
| V | -0.53 | 0.99 | -1.52 | -0.58 | 0.25 | -1.12 | -0.48 | 0.79 | 0.08 | 0.44 | 1.21 | 0.37 | -0.84 | 0.15 | 1.15 | 0.06 | 0.43 | -5.11 | 1.74 | 2.53 |
| W | -6.76 | -5.88 | -11.17 | -10.57 | -2.57 | -7.10 | -4.04 | -4.84 | -6.35 | -4.52 | -3.79 | -4.81 | -6.27 | -4.10 | -4.23 | -5.59 | -5.52 | -5.65 | 106.81 | -3.06 |
| Y | -6.36 | -5.50 | -8.68 | -8.15 | -5.37 | -6.25 | -5.91 | -5.74 | -6.94 | -5.83 | -5.19 | -6.25 | -6.66 | -5.13 | -4.13 | -6.29 | -6.20 | -6.41 | -4.24 | 115.22 |
