# Supplementary material for: Developing similarity matrices for antibody-protein binding interactions
Source: PLoS One. 2023 Oct 26;18(10):e0293606. doi: 10.1371/journal.pone.0293606 (PMC10602319; doi:10.1371/journal.pone.0293606)
Supplement: S4 Table — The trends are qualitatively similar to those for the antibody mutations. (DOCX) [file pone.0293606.s004.docx]

**Supplemental Table 4: The representative values for mutations of antigen residues calculated by Amber.** The trends are qualitatively similar to those for the antibody mutations.

|  | A | C | D | E | F | G | H | I | K | L | M | N | P | Q | R | S | T | V | W | Y |
| --- | --- | --- | --- | --- | --- | --- | --- | --- | --- | --- | --- | --- | --- | --- | --- | --- | --- | --- | --- | --- |
| A | 39.65 | -2.88 | -4.51 | -3.65 | -2.14 | -4.28 | -2.14 | -2.71 | 1.47 | -4.49 | -2.50 | -0.18 | -4.31 | -2.22 | 4.18 | -1.93 | -1.91 | -4.19 | -0.90 | -0.37 |
| C | -4.54 | 66.34 | -3.57 | -2.10 | -3.54 | -4.42 | -3.17 | -2.91 | -1.77 | -3.80 | -2.77 | -2.21 | -5.35 | -2.12 | -3.14 | -4.58 | -4.42 | -4.33 | -3.25 | -4.36 |
| D | -12.14 | -11.80 | 217.42 | -7.84 | -11.25 | -12.66 | -10.98 | -11.87 | -11.89 | -11.96 | -12.05 | -10.64 | -12.22 | -11.09 | -10.90 | -11.90 | -11.32 | -11.73 | -11.68 | -11.48 |
| E | -13.83 | -13.48 | -8.97 | 243.68 | -12.87 | -14.04 | -12.39 | -13.69 | -12.92 | -13.42 | -12.54 | -12.96 | -13.64 | -11.89 | -11.63 | -13.45 | -13.19 | -13.60 | -12.36 | -12.83 |
| F | -10.21 | -9.02 | -8.87 | -7.98 | 165.07 | -10.83 | -8.91 | -9.01 | -6.84 | -9.05 | -7.60 | -8.27 | -10.05 | -7.26 | -6.91 | -9.10 | -8.86 | -9.67 | -8.58 | -8.06 |
| G | -5.93 | -4.42 | -5.45 | -4.01 | -6.43 | 95.39 | -5.35 | -6.61 | -3.11 | -5.29 | -5.41 | -4.71 | -7.87 | -3.24 | -1.85 | -4.74 | -5.01 | -6.85 | -3.90 | -5.22 |
| H | -7.50 | -6.23 | -4.60 | -4.88 | -4.96 | -7.71 | 101.24 | -5.22 | -4.44 | -5.63 | -5.03 | -5.46 | -6.98 | -4.91 | -2.15 | -5.84 | -5.29 | -6.24 | -3.50 | -4.69 |
| I | -5.09 | -3.26 | -4.27 | -5.62 | -4.97 | -5.52 | -3.71 | 74.67 | -1.81 | -4.22 | -3.47 | -3.43 | -5.82 | -1.71 | -0.87 | -3.97 | -4.55 | -3.79 | -4.69 | -3.91 |
| K | -12.93 | -12.78 | -13.44 | -12.83 | -12.46 | -13.66 | -12.72 | -12.65 | 236.03 | -12.81 | -12.15 | -12.29 | -13.13 | -11.45 | -8.89 | -12.69 | -12.29 | -12.63 | -11.97 | -12.29 |
| L | -7.28 | -6.97 | -8.03 | -5.22 | -5.16 | -8.31 | -5.59 | -6.69 | -4.60 | 117.17 | -6.25 | -5.02 | -8.35 | -4.39 | -1.75 | -7.31 | -7.10 | -7.12 | -6.67 | -5.36 |
| M | -7.50 | -7.58 | -9.04 | -5.84 | -5.56 | -8.61 | -8.18 | -6.09 | -4.21 | -5.71 | 123.37 | -6.15 | -8.09 | -5.00 | -2.91 | -7.24 | -6.91 | -6.60 | -6.57 | -5.61 |
| N | -8.08 | -7.14 | -6.79 | -5.29 | -7.36 | -8.42 | -7.38 | -7.15 | -4.50 | -7.41 | -7.53 | 128.19 | -7.68 | -5.57 | -2.74 | -6.77 | -6.55 | -7.68 | -7.12 | -7.00 |
| P | -5.05 | -4.03 | -2.95 | -3.33 | -3.08 | -5.67 | -3.82 | -3.99 | -0.57 | -3.29 | -3.56 | -1.57 | 55.47 | -2.11 | 2.13 | -2.11 | -3.33 | -3.57 | -1.88 | -3.68 |
| Q | -9.13 | -8.70 | -6.43 | -6.07 | -7.55 | -9.60 | -7.40 | -7.93 | -5.16 | -8.49 | -7.18 | -6.30 | -8.36 | 144.41 | -5.42 | -8.56 | -7.46 | -8.77 | -8.09 | -7.83 |
| R | -14.77 | -14.14 | -15.25 | -14.08 | -14.16 | -15.43 | -14.02 | -13.95 | -11.13 | -14.51 | -13.25 | -13.19 | -14.38 | -13.16 | 265.14 | -13.95 | -14.08 | -14.26 | -14.06 | -13.39 |
| S | -6.35 | -5.84 | -4.22 | -3.73 | -6.50 | -6.76 | -5.26 | -5.80 | -3.61 | -6.48 | -4.70 | -3.40 | -6.72 | -3.58 | -1.55 | 94.82 | -3.98 | -5.78 | -4.81 | -5.74 |
| T | -8.03 | -6.85 | -7.44 | -5.02 | -7.44 | -9.33 | -6.51 | -7.29 | -4.50 | -7.40 | -6.47 | -5.49 | -8.22 | -5.41 | -2.89 | -6.39 | 124.55 | -7.60 | -6.30 | -5.98 |
| V | -3.54 | -2.73 | -0.64 | -2.72 | -2.45 | -4.43 | -2.54 | -2.71 | -1.49 | -4.11 | -2.29 | -2.98 | -3.96 | -1.28 | -2.28 | -1.78 | -2.74 | 49.98 | -2.58 | -2.75 |
| W | -8.16 | -7.71 | -9.19 | -7.61 | -7.07 | -8.88 | -6.91 | -7.49 | -6.91 | -7.33 | -7.31 | -7.30 | -8.63 | -7.21 | -4.84 | -8.04 | -7.68 | -8.09 | 143.68 | -7.33 |
| Y | -7.74 | -8.30 | -7.91 | -5.34 | -6.87 | -8.60 | -7.16 | -6.97 | -5.73 | -8.21 | -7.51 | -7.08 | -8.64 | -7.16 | -5.10 | -7.13 | -6.98 | -8.89 | -6.32 | 137.60 |
