# Supplementary material for: Developing similarity matrices for antibody-protein binding interactions
Source: PLoS One. 2023 Oct 26;18(10):e0293606. doi: 10.1371/journal.pone.0293606 (PMC10602319; doi:10.1371/journal.pone.0293606)
Supplement: S5 Table — The strongest outliers in this data compared to the other tables are the number of favorable mutations for glycine and serine to nonpolar and aromatic residues. (DOCX) [file pone.0293606.s005.docx]

**Supplemental Table 5: The representative values for mutations of antigen residues calculated by Rosetta.** The strongest outliers in this data compared to the other tables are the number of favorable mutations for glycine and serine to nonpolar and aromatic residues.

|  | A | C | D | E | F | G | H | I | K | L | M | N | P | Q | R | S | T | V | W | Y |
| --- | --- | --- | --- | --- | --- | --- | --- | --- | --- | --- | --- | --- | --- | --- | --- | --- | --- | --- | --- | --- |
| A | 72.00 | -0.85 | -4.71 | -5.98 | -1.12 | -4.69 | -6.28 | -0.93 | -8.06 | -2.33 | -3.08 | -5.75 | -4.48 | -3.67 | -6.11 | -5.33 | -4.63 | -1.24 | -0.09 | -2.68 |
| C | -2.61 | 114.22 | -6.53 | -7.83 | -1.10 | -4.92 | -7.28 | -3.16 | -12.33 | -2.23 | -4.20 | -4.25 | -8.01 | -8.36 | -10.32 | -6.56 | -5.22 | -2.72 | -10.67 | -5.93 |
| D | -7.79 | -7.67 | 151.94 | -5.93 | -7.26 | -9.21 | -8.87 | -7.77 | -10.90 | -6.34 | -6.64 | -8.55 | -9.39 | -7.73 | -9.51 | -8.52 | -7.56 | -7.57 | -7.17 | -7.56 |
| E | -7.63 | -6.03 | -7.06 | 138.14 | -5.53 | -8.50 | -7.89 | -5.62 | -10.22 | -5.14 | -5.35 | -8.14 | -8.63 | -6.19 | -9.89 | -9.19 | -8.44 | -5.64 | -5.87 | -7.19 |
| F | -10.27 | -10.66 | -11.98 | -12.45 | 195.29 | -11.96 | -8.94 | -8.53 | -10.51 | -9.57 | -8.21 | -10.25 | -12.46 | -10.18 | -10.44 | -11.63 | -10.28 | -8.64 | -8.13 | -10.22 |
| G | 2.27 | 1.29 | -4.18 | -2.08 | 2.22 | 18.55 | -3.50 | -0.72 | -2.75 | 1.89 | 2.91 | -2.30 | -6.88 | 0.02 | -2.59 | -4.67 | -2.83 | 0.96 | 2.39 | -0.03 |
| H | -2.44 | -2.44 | -4.98 | -5.55 | -0.92 | -4.01 | 58.11 | -1.60 | -3.46 | -2.18 | -0.86 | -5.01 | -4.49 | -4.24 | -2.61 | -5.10 | -4.03 | -1.78 | -1.01 | -1.40 |
| I | -5.72 | -4.42 | -9.57 | -8.45 | -3.27 | -8.74 | -6.53 | 117.44 | -7.64 | -3.02 | -3.88 | -7.97 | -6.80 | -7.21 | -7.18 | -8.17 | -6.95 | -4.45 | -3.43 | -4.06 |
| K | -8.18 | -7.69 | -10.89 | -10.23 | -7.51 | -8.97 | -9.61 | -7.30 | 158.55 | -6.84 | -7.11 | -9.26 | -9.04 | -8.65 | -6.81 | -9.56 | -8.37 | -7.16 | -7.31 | -8.08 |
| L | -7.20 | -6.70 | -8.19 | -11.22 | -5.98 | -9.61 | -7.40 | -4.22 | -10.64 | 145.10 | -4.23 | -7.89 | -9.25 | -7.94 | -9.33 | -10.66 | -7.14 | -4.32 | -5.56 | -7.65 |
| M | -11.87 | -11.11 | -14.80 | -15.42 | -5.97 | -14.26 | -11.52 | -11.93 | -14.08 | -7.39 | 220.21 | -10.87 | -13.09 | -11.13 | -11.10 | -15.53 | -11.55 | -9.75 | -9.02 | -9.84 |
| N | -2.39 | -2.26 | -5.37 | -5.46 | -0.57 | -5.07 | -5.37 | -0.07 | -6.97 | -1.26 | -1.78 | 64.11 | -3.58 | -2.27 | -5.87 | -6.71 | -3.54 | -1.70 | -0.78 | -3.10 |
| P | -3.75 | -1.63 | -8.12 | -8.02 | -2.01 | -5.86 | -7.09 | -1.76 | -8.06 | -1.35 | -0.64 | -6.83 | 84.32 | -6.29 | -5.08 | -8.25 | -5.04 | -1.36 | 0.77 | -3.97 |
| Q | -5.31 | -6.09 | -7.97 | -8.51 | -4.16 | -7.76 | -6.70 | -4.66 | -8.61 | -3.30 | -3.57 | -6.79 | -7.52 | 121.70 | -6.62 | -8.80 | -7.58 | -4.98 | -6.49 | -6.28 |
| R | -6.99 | -6.54 | -9.55 | -8.59 | -4.43 | -8.57 | -8.03 | -4.55 | -5.66 | -5.04 | -5.20 | -7.01 | -9.16 | -5.89 | 129.63 | -8.85 | -8.05 | -6.32 | -4.82 | -6.38 |
| S | -2.13 | 0.54 | -4.42 | -4.78 | 2.05 | -2.73 | -2.76 | -1.25 | -3.68 | 0.18 | 2.29 | -5.00 | -4.65 | -2.47 | -6.05 | 35.30 | -2.81 | 0.41 | -0.32 | 2.27 |
| T | -4.46 | -5.60 | -8.73 | -9.80 | -3.06 | -7.87 | -9.11 | -3.97 | -9.99 | -4.15 | -4.80 | -9.36 | -9.70 | -5.96 | -7.62 | -9.01 | 126.31 | -5.45 | -2.97 | -4.73 |
| V | -4.61 | -4.92 | -9.48 | -8.00 | -4.02 | -8.67 | -8.17 | -3.23 | -8.94 | -6.38 | -2.02 | -8.57 | -8.48 | -7.09 | -8.47 | -10.51 | -6.89 | 129.83 | -5.51 | -5.89 |
| W | -13.89 | -13.04 | -14.65 | -12.70 | -10.07 | -13.59 | -12.96 | -9.60 | -13.14 | -9.42 | -9.49 | -13.20 | -13.90 | -14.17 | -14.92 | -15.21 | -13.50 | -12.56 | 243.51 | -13.53 |
| Y | -8.61 | -8.12 | -8.69 | -7.26 | -4.53 | -8.80 | -8.04 | -6.16 | -8.43 | -6.04 | -5.66 | -9.77 | -10.64 | -6.00 | -7.16 | -10.17 | -7.30 | -7.69 | -6.25 | 145.34 |
